# Engineering multimode interactions in circuit quantum acoustodynamics

**DOI:** 10.1038/s41567-023-02377-w

**Published:** 2024-01-25

**Authors:** Uwe von Lüpke, Ines C. Rodrigues, Yu Yang, Matteo Fadel, Yiwen Chu

**Affiliations:** 1 Department of Physics, ETH Zürich, Zurich, Switzerland; 2 Quantum Center, ETH Zürich, Zürich, Switzerland

**Keywords:** Quantum information, Quantum mechanics, Qubits

## Abstract

In recent years, important progress has been made towards encoding and processing quantum information in the large Hilbert space of bosonic modes. Mechanical resonators have several practical advantages for this purpose, because they confine many high-quality-factor modes into a small volume and can be easily integrated with different quantum systems. However, it is challenging to create direct interactions between different mechanical modes that can be used to emulate quantum gates. Here we demonstrate an in situ tunable beamsplitter-type interaction between several mechanical modes of a high-overtone bulk acoustic-wave resonator. The engineered interaction is mediated by a parametrically driven superconducting transmon qubit, and we show that it can be tailored to couple pairs or triplets of phononic modes. Furthermore, we use this interaction to demonstrate the Hong–Ou–Mandel effect between phonons. Our results lay the foundations for using phononic systems as quantum memories and platforms for quantum simulations.

## Abstract (web summary)

Quantum gates require controlled interactions between different degrees of freedom. A tunable coupling has now been demonstrated between the phonon modes of a mechanical resonator designed for storing and manipulating quantum information.

## Main

Mechanical degrees of freedom are a particularly interesting quantum platform, as they involve the collective motion of massive particles, can have long coherence times and can be combined with many other quantum systems^1^. Circuit quantum acoustodynamics (cQAD) systems, where a superconducting qubit is coupled to gigahertz-frequency acoustic modes, have recently been engineered^2–4^ and used to demonstrate the generation and measurement of non-trivial quantum states^4–8^ and entanglement between mechanical modes^9^. Due to the small mode volumes, low crosstalk and high coherence times of acoustic modes, cQAD devices have become the target platform of recent proposals for the realization of a quantum random-access memory^10^ as well as fault-tolerant quantum computing architectures^11,12^. In particular, cQAD devices that incorporate high-overtone bulk acoustic-wave resonators (HBARs) can take advantage of the HBAR’s large effective mass and multimode properties, making them excellent platforms for the implementation of bosonic quantum simulations^13–15^, bosonic encodings^16,17^, quantum metrology applications^18^ and fundamental studies of quantum mechanical interference phenomena between phonons^19–21^.

An important yet currently missing tool for the realization of these applications is the generation of a phononic iSWAP gate, which is an operation that allows for a direct exchange of quanta between mechanical modes. This can be engineered via a beamsplitter interaction, a coupling mechanism that has already been studied between photonic modes^22,23^, in optomechanical systems^24^, in trapped ions^25^, between mechanical resonators in the classical regime^19,26^ and between travelling mechanical waves^21^. When brought to the quantum regime, this phononic beamsplitter interaction will not only become a building block of quantum computing architectures^10–12^ but will also offer new possibilities for the simulation of complex quantum systems and the phononic realization of quantum-optics-type experiments that have so far been mostly explored with photonic systems.

In this work, we demonstrate a beamsplitter interaction between multiple phonon modes of an HBAR coupled to a superconducting transmon qubit. We create this interaction by applying two off-resonant drives on the qubit^27^ such that it acts as a nonlinear mixing element. We first study the effects of this bichromatic driving through qubit spectroscopy, observe the generation of multiple sidebands and show how these sidebands mediate the desired beamsplitter coupling. Having realized this interaction, we then perform time-domain experiments to demonstrate both iSWAP and $\sqrt{{\rm{i}}{{{\rm{SWAP}}}}}$ gates, subsequently using the latter to demonstrate entanglement between two acoustic overtone modes of our HBAR. Furthermore, by choosing another parameter regime, we create an interference between three phononic modes and explore the multimode dynamics governing the system. Finally, we utilize the beamsplitter interaction to exchange multiple excitations between the modes and observe the Hong–Ou–Mandel interference^21,22,25,28–30^ between macroscopic mechanical modes.

The device used in this work is a cQAD system where a superconducting qubit is flip-chip bonded to an HBAR^7^. The qubit is a three-dimensional transmon with a frequency of *ω*_*q*_ = 2π × 5.97 GHz, an energy relaxation time of *T*_1_ = 9.5 µs, a Ramsey decoherence time of ${T}_{2}^{\,* }$ = 7.2 µs and an anharmonicity *α* = 2π × 218 MHz. The longitudinal free spectral range (FSR) of the HBAR is approximately 2π × 12.63 MHz, and the two subsystems are coupled through a piezoelectric transducer that mediates a Jaynes–Cummings (JC) interaction with a coupling strength of *g*_*m*_ = 2π × 257 kHz. The device is housed in a three-dimensional aluminium cavity, which we use to both shield the qubit from its environment and read its state via the dispersive interaction between the qubit and the cavity. Supplementary Table I provides a full list of system parameters.

Although the cQAD device used in this work has been previously studied in both dispersive^5^ and resonant coupling regimes^6^, here we focus on direct multimode interactions that arise when two parametric drives are applied to the qubit. The Hamiltonian of our system in the presence of these drives is given by

1 $$\begin{array}{rcl}H&=&{\omega }_{{q}}{q}^{{\dagger} }q-\frac{\alpha }{2}{{q}^{{\dagger} }}^{2}{q}^{2}\\ &+&\mathop{\sum}\limits_{m}\left[{\omega }_{m}{m}^{{\dagger} }m+{g}_{m}({m}^{{\dagger} }q+m{q}^{{\dagger} })\right]+{H}_{{{{\rm{qd}}}}},\end{array}$$

where we assume *g*_*m*_ to be real. Here the first two terms describe the qubit as an anharmonic mode with lowering operator *q*. The sum over phonon modes *m* = *a*, *b*, *c*… with frequencies *ω*_*m*_ and lowering operators *m* includes their energies as well as their JC interaction with the qubit. The last term, given by ${H}_{{{{\rm{qd}}}}}=\left({\varOmega }_{1}{{\rm{e}}}^{-{\rm{i}}{\omega }_{1}t}+{\varOmega }_{2}{{\rm{e}}}^{-{\rm{i}}{\omega }_{2}t}\right){q}^{{\dagger} }+{{{\rm{h.c.}}}}$, describes two off-resonant microwave drives applied to the qubit with frequencies *ω*_1_ = *ω*_*q*_ + 2π × 492.5 MHz and *ω*_2_ ≈ *ω*_1_ + FSR. The drives, together with two modes *a* and *b*, can participate in a four-wave mixing process mediated by the Josephson nonlinearity of the superconducting qubit^10,27,31^. In particular, when the resonance condition *Δ*_21_ ≡ *ω*_2_ − *ω*_1_ = *ω*_*b*_ − *ω*_*a*_ is satisfied, equation (1) leads to a bilinear coupling between the phonon modes. Even though this picture is quantitatively accurate for large phonon–phonon detunings and small drive strengths, we now present a framework that extends this picture to address the case of large drive strengths and small phonon–phonon detunings. Furthermore, our analysis readily lends itself to systems with many bosonic modes by explicitly considering processes involving multiple drive photons.

**Fig. 1:**
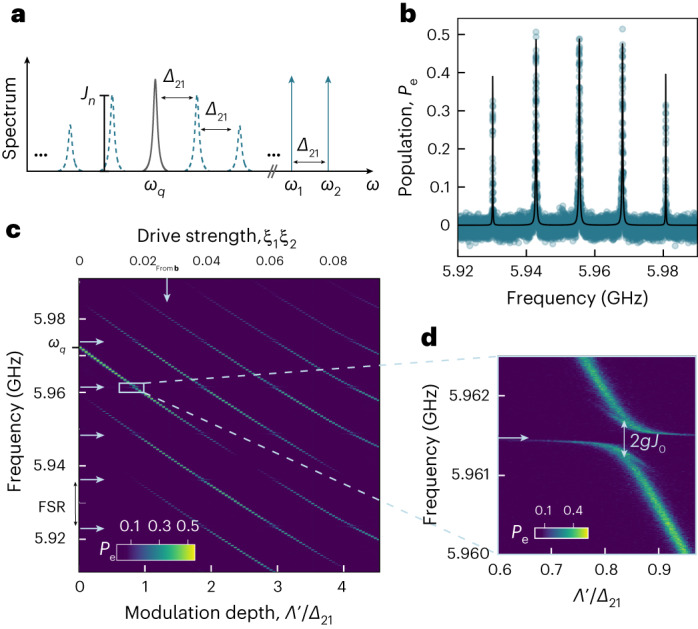
Effects of bichromatic driving on a transmon qubit coupled to an HBAR. **a**, Schematic of the spectrum of a bichromatically driven qubit. The blue vertical lines represent the two drives, the black Lorentzian peak represents the qubit resonance and the dashed Lorentzian peaks in blue represent the generated sidebands with amplitudes *J*_*n*_ for the *n*th sideband. **b**, Qubit population *P*_e_ during spectroscopy for a drive strength of *ξ*_1_*ξ*_2_ ≈ 0.0274. The circles are the data and the black line is a theoretical curve (Supplementary Section II) for the measured qubit population *P*_e_ when sweeping a probe tone over the qubit sidebands. **c**, Qubit spectroscopy for different values of modulation depth *Λ*′/*Δ*_21_. The top *x* axis indicates the corresponding drive strength *ξ*_1_*ξ*_2_. The vertical arrow indicates the linescan shown in **b** and the horizontal arrows indicate the phonon-mode frequencies of the HBAR. **d**, Zoomed-in view of one of the qubit–phonon anti-crossings in **c**. Source data

We first consider only the effect of the drives on the qubit itself. Due to transmon anharmonicity, going into the displaced frame of the drives results in a modulated a.c. Stark shift of the qubit frequency given by (Supplementary Section V)

2 $${H}_{{{{\rm{Stark}}}}}=\left[-2\alpha ({\xi }_{1}^{2}+{\xi }_{2}^{2})-4\alpha {\xi }_{1}{\xi }_{2}\cos ({\Delta }_{21}t)\right]{q}^{{\dagger} }q,$$

with the dimensionless drive strengths *ξ*_*j*_ = *Ω*_*j*_/*Δ*_*j*_, where *Δ*_*j*_ = *ω*_*j*_ − *ω*_*q*_ for *j* ∈ {1, 2}. This shift has a time-independent as well as a time-dependent contribution, the latter arising from the beating between the two drives, which modulates the qubit frequency with *Δ*_21_. As usual for a frequency-modulated system^32–34^ (Fig. 1a), this gives rise to the appearance of multiple qubit sidebands separated by *Δ*_21_, whose amplitudes are given by ${J}_{n}\left(\frac{\Lambda }{{\Delta }_{21}}\right)$. Here *J*_*n*_(*x*) is the Bessel function of the first kind for a given sideband number *n*, and *Λ* = −4*αξ*_1_*ξ*_2_. We note that due to the interplay of the parametric drives with the third energy level of the qubit, *H*_Stark_ acquires a correction, which we derive using time-independent perturbation theory (Supplementary Section III). In the following, we use the corrected value for the modulation depth, which we label as *Λ*′. Furthermore, we will use the shorthand ${J}_{n}={J}_{n}\left(\frac{{\Lambda }^{{\prime} }}{{\Delta }_{21}}\right)$.

We experimentally confirm these effects via two-tone spectroscopy. Specifically, we sweep a weak probe signal across the qubit frequency with the off-resonant drives turned on and subsequently measure the resulting qubit population using dispersive readout. As expected, we find multiple resonances separated by *Δ*_21_ with different peak heights, which are the qubit sidebands described above (Fig. 1b). The measured steady-state population of the qubit is quantitatively described in the same way as in a regular qubit spectroscopy experiment^35^, with the probe strength adjusted by the sideband amplitude (Fig. 1b, continuous black line). After repeating the measurement for a range of parametric drive strengths *ξ*_1_*ξ*_2_ (*ξ*_1_ = *ξ*_2_), we find the result shown in Fig. 1c, where we observe multiple diagonal lines spaced in frequency by *Δ*_21_ and with varying intensities. These qubit sidebands shift to lower frequencies with increasing drive power, as expected from the Stark shift described by the first term in equation (2).

The JC interaction between the driven qubit and phonon modes results in anti-crossings where the frequency of a sideband matches that of a phonon mode (Fig. 1c,d). However, the effective qubit–phonon coupling strength is scaled by the amplitude of the sideband closest to the phonon mode. Therefore, the gap of the anti-crossing will be reduced from 2*g*_*m*_ to 2*J*_*n*_*g*_*m*_, as indicated for *n* = 0 (Fig. 1d).

In the dispersive regime, where all the qubit sidebands and phonon modes are far detuned, it is useful to enter the interaction picture of the sideband-mediated qubit–phonon coupling via the Schrieffer–Wolff transformation^36^. After applying the rotating-wave approximation, we can identify two effects in the resulting effective Hamiltonian. First, there is a frequency shift in the phonon modes, due to their hybridization with the qubit^37^, such that the phonon frequency in the presence of the driven qubit is *ω*_*m*_ + *δ*_*m*_ with

3 $${\delta }_{m}={g}_{m}^{2}\mathop{\sum}\limits_{n}\frac{{J}_{n}^{2}}{{\tilde{\Delta }}_{m}-n{\Delta }_{21}},$$

where ${\tilde{\Delta }}_{m}={\omega }_{m}-{\tilde{\omega }}_{q}$ is the detuning between phonon mode *m* and the Stark-shifted qubit. We see that a phonon mode’s frequency shift is dominated by the sideband for which the denominator in equation (3) is the smallest. Second, although the Schrieffer–Wolff transformation typically eliminates the JC coupling term between the qubit and phonons, in our case, it also gives rise to phonon–phonon coupling terms. For example, the coupling between two neighbouring phonon modes *b* and *c* is given by *g*_*bc*_(*b*^†^*c* + *bc*^†^), with

4 $${g}_{{{{{bc}}}}}={g}_{b}{g}_{c}\mathop{\sum}\limits_{n}\frac{{J}_{n}{\,J}_{n+1}}{{\tilde{\Delta }}_{b}-n{\Delta }_{21}},$$

when *Δ*_21_ = *ω*_*c*_ − *ω*_*b*_ + *δ*_*c*_ − *δ*_*b*_, such that this term remains after the rotating-wave approximation. Here *δ*_*b*,*c*_ refer to the frequency shift of phonons *b* and *c* as described by equation (3). Similarly, the next-nearest-neighbouring phonon modes *a* and *c* experience a coupling of *g*_*ac*_(*a*^†^*c* + *ac*^†^), with

5 $${g}_{{{{{ac}}}}}={g}_{a}{g}_{c}\mathop{\sum}\limits_{n}\frac{{J}_{n}{\,J}_{n+2}}{{\tilde{\Delta }}_{a}-n{\Delta }_{21}},$$

when 2*Δ*_21_ = *ω*_*c*_ − *ω*_*a*_ + *δ*_*c*_ − *δ*_*a*_.

The numerator of equation (4), which contains the product of two successive Bessel functions, represents the physical process of the qubit converting one photon between the parametric drives. The frequency conversion of the drive photons compensates for the energy difference between the phonon modes, making the beamsplitter interaction resonant. Interestingly, the effective coupling strength for this process does not become larger monotonically with increasing drive strengths *ξ*_1_*ξ*_2_. Instead, the speed of the single-photon conversion is reduced in favour of multiphoton processes, for example, converting two drive photons to bridge the energy gap between the phonon modes with a frequency difference of 2*Δ*_21_ (equation (5)). Supplementary Sections II and IV provide a more detailed derivation of the different transformations and their effects on the system Hamiltonian.

**Fig. 2:**
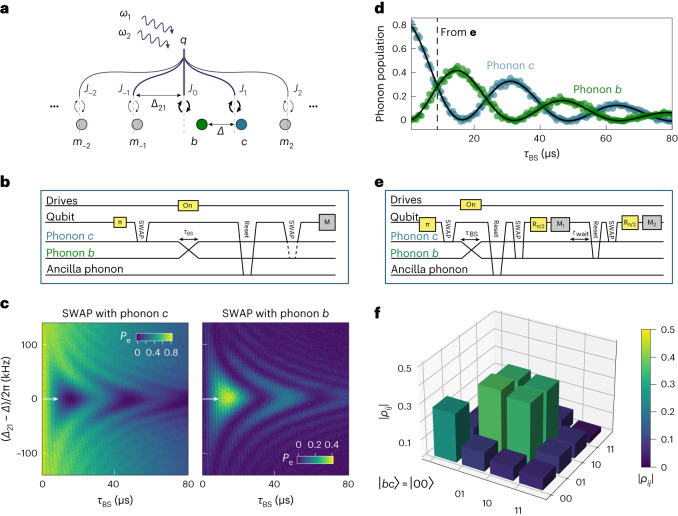
Beamsplitter interaction between two acoustic modes. **a**, Schematic of the beamsplitter coupling between two mechanical modes *b* and *c* mediated by the qubit sidebands. The frequency difference between the drives is given by *Δ*_21_, whereas the acquired unique spacing between the two neighbouring modes of interest is given by *Δ*. **b**, Pulse sequence used in the experiment shown in **c**, where the two modes are subsequently measured in different sequences. **c**, Phonon population versus detuning (*Δ*_21_ − *Δ*) and interaction time *τ*_BS_. We perform the pulse sequence described in **b** as the drive frequency *ω*_2_ is changed and the population is read in either mode *c* (left plot) or mode *b* (right plot). The horizontal white arrow indicates the curves shown in **d**. **d**, Rabi oscillations between the two mechanical modes when *Δ*_21_ = *Δ*. The vertical dashed line shows the interaction time *τ*_BS_ = 8.0 µs at which the tomography experiment in **e** was performed. The black lines are fits to a decaying sinusoidal function. **e**, Pulse sequence used for the tomography experiment shown in **f**. **f**, Reconstructed density matrix for a joint phonon state after a 50:50 beamsplitter interaction. Both the colours and height indicate the magnitude of the matrix elements. Source data

The dependence of the qubit sidebands on the Bessel functions is what allows us to choose different combinations of coupling strengths between the phonon modes and frequency shifts throughout this work. Naively, it might seem that due to the equal frequency spacing of the phonon modes, one cannot choose interactions between only a subset to be resonant. However, this is not the case. For instance, by choosing an appropriate modulation depth *Λ*′/*Δ*_21_, we can choose the amplitude of *J*_0_ to be larger than those of the neighbouring sidebands, namely, *J*_1_ and *J*_−1_. According to equation (3), the phonon mode closest to the zeroth sideband will shift by a larger amount $(\propto {J}_{0}^{2})$ than the adjacent phonon modes $(\propto {J}_{1}^{2},\,{J}_{-1}^{2})$, giving rise to a unique frequency spacing between the two phonon modes equal to *Δ*_21_ and promoting a beamsplitter interaction between them (Fig. 2a). If, on the other hand, we choose a regime where *J*_0_ = *J*_1_ = − *J*_−1_, the three phonon modes *a*, *b* and *c* adjacent in frequency to the *n* = −1, 0 and 1 sidebands, respectively, will be equally shifted, promoting beamsplitter interactions between these three modes. Note that in the latter case, the next-nearest-neighbour modes *a* and *c* are coupled via a two-photon conversion described by equation (5).

We now experimentally investigate the first case of coupling between only the two modes *b* and *c* (Fig. 2a). By choosing appropriate drive strengths *ξ*_1,2_, we set the modulation depth to *Λ*′/*Δ*_21_ = 0.610 ± 0.001 such that *J*_0_ = 0.91 ± 0.01 and *J*_1_ = 0.29 ± 0.01. Here the errors are propagated from uncertainties in the independent measurement of system parameters (Supplementary Table I). Our experimental protocol starts with swapping an excitation from the qubit into mode *c* using the resonant JC interaction. Note that we use a third microwave drive, far detuned from the parametric drives, to independently adjust the frequency of the qubit for this swap operation and to compensate the Stark shift of the qubit from the parametric drives during the beamsplitter interaction to set ${\tilde{\varDelta }}_{{b}}$ = 2π × 1.0 MHz ± 17 kHz. We then turn on the parametric drives for a variable time *τ*_BS_ (Fig. 2b). Afterwards, the qubit has a finite excited-state population due to the off-resonant drives. We reset the qubit to its ground state by swapping its residual population to an ancillary phonon mode detuned by several FSRs from the modes of interest^6^. Finally, we swap the excitation from mode *b* or *c* into the qubit and measure its excited-state population.

Repeating this experiment for different values of *Δ*_21_, we observe the expected chevron pattern produced by a beamsplitter-type interaction between the two modes (Fig. 2c). Here we vary *Δ*_21_ by only about ±1%, such that we can treat the modulation depth as constant. When *Δ*_21_ matches the unique detuning between the two modes *Δ*, we satisfy the resonance condition for the four-wave mixing process, and the exchange of quanta between the modes becomes most efficient. This occurs for a modulation frequency of (*Δ*_21_ − FSR) = −2π × 44 kHz, which matches our prediction from equation (3). We plot the phonon-mode populations for *Δ*_21_ = *Δ* (Fig. 2d) and fit them each to a decaying oscillation, yielding a beamsplitter coupling rate of *g*_bc_ = 2π × 15.6 ± 0.1 kHz. Note that the contrast for the oscillation in phonon mode *b* is slightly lower than that for phonon mode *c*. This is a result of the different decay rates between the two phonon modes, as well as a small but finite leakage to the next phonon mode, namely, *m*_−1_ (Fig. 2a). The microscopic origin of the different decay rates for different HBAR modes is a subject of ongoing research^38^.

At the time *τ*_BS_ = π/4*g*_BS_ = 8.0 μs (Fig. 2d, dashed line), the interaction becomes a 50:50 beamsplitter or $\sqrt{{\rm{i}}{{{\rm{SWAP}}}}}$ gate, which creates an entangled state between the two phonon modes. We experimentally confirm this by performing two-qubit state tomography on the resulting state (Fig. 2e). Here, in contrast to the data shown in Fig. 2c,d, we measure the observables of both phonon modes in the same sequence, thereby accessing joint two-mode observables necessary for full-state tomography. To quantify the created entanglement, we compute an overlap of the reconstructed density matrix with the maximally entangled state $\left\vert bc\right\rangle =(\left\vert 01\right\rangle +{{\rm{e}}}^{{\rm{i}}\phi }\left\vert 10\right\rangle )/\sqrt{2}$ of *F*_Bell_ = 0.69 ± 0.01, with *ϕ* chosen to optimize *F*_Bell_. This confirms the presence of entanglement between the two phonon modes. We attribute the difference between the reconstructed density matrix and the maximally entangled state to phonon decay during the $\sqrt{{\rm{i}}{{{\rm{SWAP}}}}}$ gate and an imperfect state preparation of the initial Fock state in mode *c*. Supplementary Section VI provides details on the tomography procedure.

**Fig. 3:**
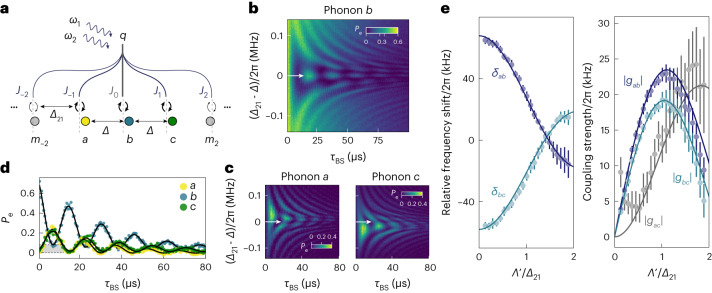
Engineering a multimode coupling by tuning the parametric drive power. **a**, Schematic of beamsplitter coupling between three modes. **b**, Final phonon *b* occupation versus detuning (*Δ*_21_ − *Δ*) and interaction time *τ*_BS_. **c**, Final phonon *a* (*c*) population versus detuning and interaction time. **d**, Linescans of the individual phonon populations versus *τ*_BS_ for *Δ*_21_ = *Δ*, as indicated in **b** and **c** with the horizontal white arrows. The black lines are fits to a decaying sinusoidal function and the grey-shaded area indicates the offset of the residual phonon *b* occupation from zero. **e**, Relative frequency shifts and absolute coupling strengths between different phonon modes versus modulation depth *Λ*′/*Δ*_21_. The data (circles) were extracted from fitting the data like the ones displayed in **b** and **c** for various values of *Λ*′/*Δ*_21_. The theory curves (full lines) are computed using equations (3)–(5). The error bars are extracted on the basis of a 5% induced change on the fitting residuals. Supplementary Section VII provides more details on the fitting routine and theory description of our multimode coupling as a three-level system. Source data

Having demonstrated a beamsplitter interaction between the two phonon modes, we now move on to create simultaneous interactions between three modes. To that end, we tune the modulation depth to *Λ*′/*Δ*_21_ = 1.430 ± 0.003 such that *J*_0_ = *J*_1_ = −*J*_−1_ = 0.55 ± 0.01. In this regime, phonon modes *a*, *b* and *c* are equally shifted such that *Δ*_*cb*_ = *Δ*_*ba*_ ≡ *Δ*. This is schematically shown in Fig. 3a. In this case, phonon-mode pairs (*b*, *c*) and (*a*, *b*) are coupled via equation (4), whereas the mode pair (*a*, *c*) is coupled via equation (5), with ∣*g*_*ab*_∣ ≈ ∣*g*_*bc*_∣ ≈ ∣*g*_*ac*_∣.

To explore the dynamics of this three-mode coupling scheme, we extend the experiment presented in Fig. 2. Specifically, we load an excitation into phonon mode *b* and turn on the parametric drives, thereby activating beamsplitter interactions between all the three modes, and finally measure their population. As before, we sweep the interaction time *τ*_BS_ and the modulation frequency *Δ*_21_, with ${\tilde{\varDelta }}_{b}$ = 2π × 1.0 MHz ± 17 kHz. The results are shown in Fig. 3b,c. Although they show the expected qualitative aspects of the excitation swapping between all the three modes, we observe two interesting features. First, when *Δ*_21_ = *Δ*, the initial excitation in mode *b* flows to modes *a* and *c* with approximately equal rates (Fig. 3d). However, the excitation does not fully swap to modes *a* and *c*, which is visible from the reduced oscillation contrast (Fig. 3d, grey-shaded area). Although counterintuitive at first, this is the expected behaviour of a three-mode system with coupling between all the mode pairs. The coupling between modes *a* and *c* hybridizes them into new normal modes with frequencies shifted by the coupling strength. As a result, the coupling between these normal modes and mode *b* is no longer resonant, resulting in the reduced oscillation contrast we observe. We note that the frequency of the population exchange observed in Fig. 3d, namely, 2π × 64 ± 1.5 kHz, is in good agreement with theoretical calculations.

The second observation is that the data in Fig. 3c for mode *a* are approximately the mirror image of mode *c* with respect to *Δ*_21_ − *Δ* = 0. For instance, when *Δ*_21_ − *Δ* > 0 (*Δ*_21_ − *Δ* < 0), the initial excitation in mode *b* predominantly flows to mode *a* (*c*). Although the roles of modes *a* and *c* are symmetric when *Δ*_21_ = *Δ*, this symmetry is broken away from the resonance condition due to the coupling between modes *a* and *c* and the resulting normal-mode splitting. Supplementary Section VIII presents a detailed explanation for both these effects.

Although we present experimental details on two interesting values of modulation depth, we note that we can tune from one regime to the other by changing the drive powers, thereby observing a gradual change in both coupling strength and relative detuning (Fig. 3e). To acquire the effective interaction strengths between the three modes as well as their respective phonon frequency shifts, we perform the experiment shown in Fig. 3b,c for different values of *ξ*_1_*ξ*_2_, thereby varying *Λ*′/*Δ*_21_. We then fit the measured phonon populations to a set of coupled equations of motion with beamsplitter couplings *g*_*mk*_ and relative phonon detunings *δ*_*mk*_ as free parameters (*m*, *k* ∈ {*a*, *b*, *c*}). Supplementary Section VII provide details on the fitting procedure. The fit results are plotted alongside equations (3)–(5) with no free parameters (Fig. 3e) and show good agreement between experiment and theory. The observed difference between ∣*g*_*ab*_∣ and ∣*g*_*bc*_∣ is a result of the different relative contributions from the sidebands in equation (4) depending on the position of the phonon modes involved. In particular, the observed reduction in ∣*g*_*ab*_∣ and ∣*g*_*bc*_∣ for larger modulation depths, as well as the accompanying increase in ∣*g*_*ac*_∣, are well captured by theory. We emphasize that previous works have only investigated a much smaller range of modulation depths; therefore, these effects were not evident^16,27,31,39^.

**Fig. 4:**
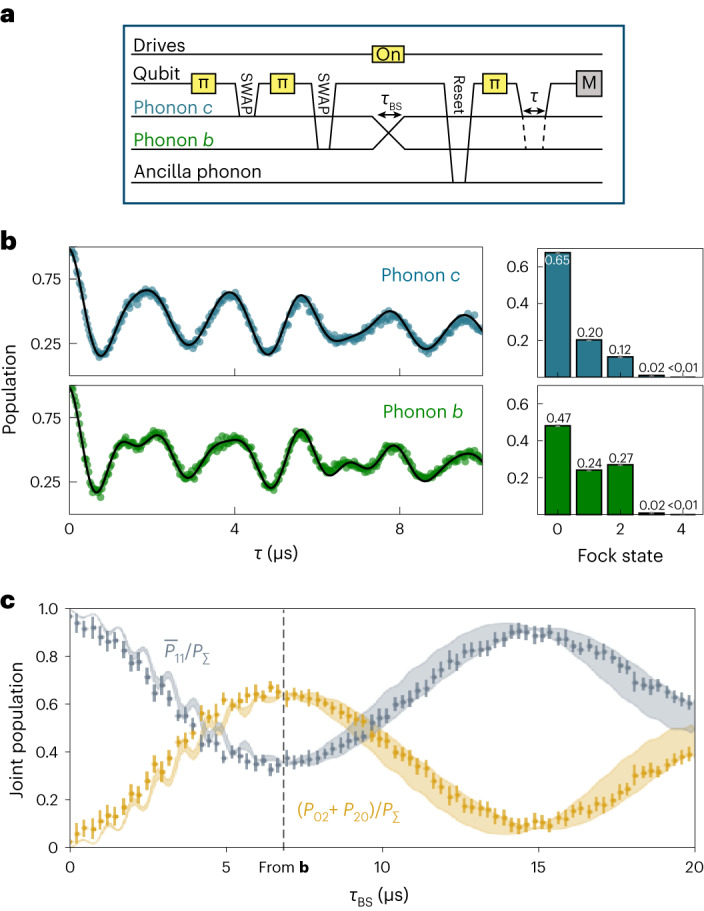
Observation of the Hong–Ou–Mandel effect between two phonon modes. **a**, Pulse sequence used in the experiment where the two modes are individually measured in different sequences. The regime addressed here is the same as that in Fig. 2, that is, a two-mode coupling between phonons *c* and *b*. **b**, Rabi oscillations between phonon modes *c* (*b*) and the qubit in the top (bottom) plot for 82 values of the resonant interaction duration *τ*. The circles are the data and the black lines are fits. The extracted Fock-state populations for each mode is shown in the histograms on the right side. The vertical error bars on the histograms (grey) account for one standard deviation of the fit uncertainty and are below 1%. **c**, Normalized joint phonon population for different interaction times *τ*_BS_. The dots are the data and the shaded areas are the result of simulation results of the full system Hamiltonian in equation (1) accounting for 3% deviation in *g*_*m*_. The error bars on the data points include higher Fock-state populations and one standard deviation of the fit uncertainties propagated from the data shown in **b**, and the dashed vertical line indicates the data shown in **b**. Source data

So far, we have studied the two- and three-mode coupling regimes for the particular case where a single phononic quantum is shared between all of the participating modes. We now investigate the interplay of two quanta during a beamsplitter operation. We first create a |*cb*〉 = |11〉 state in modes *b* and *c* by repeatedly exciting the qubit and swapping its excitation into each mode^7^. We then turn on the two-mode beamsplitter interaction and subsequently measure the resulting phonon Fock-state distributions of either mode by monitoring the qubit population during a resonant qubit–phonon JC interaction, as shown in previous work^7^ (Fig. 4a). As an example, the results for a beamsplitter time of *τ*_BS_ = 6.7 μs are shown in Fig. 4b. Here, to optimize the coupling strength and reduce the residual JC interaction with the qubit, we use a slightly larger qubit–phonon detuning of ${\tilde{\Delta }}_{b}$ = 2π × 1.2 MHz ± 17 kHz and modulation depth of *Λ*′/*Δ*_21_ = 0.850 ± 0.002, resulting in *g*_*bc*_ = 2π × 18.5 ± 0.8 kHz.

The Hong–Ou–Mandel effect predicts that the outcome of this experiment should depend on whether or not the two phonons are distinguishable. If they are, no interference between them will occur and the excitations will be equally shared between the two phonon modes. On the other hand, if they are indistinguishable, both excitations will bunch in one of the two phonon modes after the beamsplitter. To experimentally confirm this, we compare the probability of the bunched (*P*_20_ + *P*_02_) with that of the anti-bunched outcome (*P*_11_). We extract the bunched outcome probability from the individual Fock distributions by assigning *P*_02_ + *P*_20_ to ${P}_{2}^{c}+{P}_{2}^{b}$, where ${P}_{2}^{c(b)}$ is the probability of finding two quanta in mode *c* (*b*). Doing so relies on the assumption that our system contains a maximum of two excitations at the start of the beamsplitter interaction and that no additional quanta are added during the sequence. This assumption is justified because the residual thermal population of the phonon modes is less than 1.6% (ref. ^40^). Under the same assumption, we can put an upper bound on the anti-bunched probability, namely, ${\bar{P}}_{11}=\min ({P}_{1}^{b},{P}_{1}^{\,c})\ge {P}_{11}$. Nevertheless, we still take into account the possibility for leakage into higher Fock states by fitting the qubit–phonon Rabi oscillations for the first five energy levels. The population contribution of these higher levels is 0.01 on average and is then included in the error bars (Fig. 4c).

In Fig. 4c, we show both ${\bar{P}}_{11}$ and *P*_20_ + *P*_02_ for various beamsplitter interaction times *τ*_BS_, normalized by the entire two-excitation subspace ${P}_{\Sigma }={P}_{20}+{P}_{02}+{\bar{P}}_{11}$. As expected, the two-excitation manifold of the phonon state in the beginning of the interaction is dominated by |11〉. After *τ*_BS_ = 6.7 µs, which corresponds to a 50:50 beamsplitter (Fig. 4c, vertical dashed line), the joint state is more probably bunched with (*P*_20_ + *P*_02_)/*P*_Σ_ = 0.622 ± 0.028.

Although we cannot straightforwardly access the joint Fock distributions of the two phonon modes in our experiment, we can do so in a master equation simulation of our system using independently measured system parameters. The results are plotted as continuous lines in Fig. 4c, showing good agreement between data and theory. The fast oscillations that can be seen for lower interaction times in both theory and experiment arise due to an off-resonant JC interaction with the qubit. This result demonstrates how two apriori distinguishable phononic quanta in modes at different frequencies are made indistinguishable by a frequency-converting coupling, which compensates for the energy difference between the two modes, thereby confirming that the lattice vibrations constituting our phonons display behaviour that cannot be classically described.

In conclusion, we have engineered a direct beamsplitter coupling between two and three distinct mechanical modes of an HBAR. We have used the two-mode interaction to create a phononic $\sqrt{{\rm{i}}{{{\rm{SWAP}}}}}$ gate, allowing us to generate entanglement between the modes and observe the Hong–Ou–Mandel effect between two phonons. In addition to our experimental data, we have also presented a theoretical model that is in good agreement with our findings. Parametrically driven beamsplitters are being actively studied for the purpose of bosonic quantum computing^16,21,39,41^. Our work explores a new regime of this interaction, where sidebands generated by a large frequency modulation depth and the conversion of more than one drive photon plays an important role. We find our beamsplitter operation to be limited in speed by the qubit–phonon coupling strength and in fidelity by the phonon lifetimes. Larger values for both these parameters have been observed^42,43^, though combining both remains a challenge. Nevertheless, on the basis of these recent developments, we expect to be able to improve our device quality in the near future.

Our results provide a fundamental building block for performing quantum-optics-type experiments with massive mechanical excitations^6^. They also address a key challenge towards realizing a mechanical quantum random-access memory by providing one of two required operations^10^, the other one being a conditional phase operation^31^. Furthermore, our technique, in principle, allows for all-to-all coupling between a large number of phononic modes, all compactly hosted within a single physical resonator. This makes our device a hardware-efficient platform for future studies of non-reciprocal interactions^19,44^ and quantum simulations with bosonic modes^13,14,45^. Finally, our current system and the concepts discussed here can potentially be extended to single- and two-mode squeezing interactions, enabling Gaussian quantum information processing using mechanical resonators^46^.

## Online content

Any methods, additional references, Nature Portfolio reporting summaries, source data, extended data, supplementary information, acknowledgements, peer review information; details of author contributions and competing interests; and statements of data and code availability are available at 10.1038/s41567-023-02377-w.

### Supplementary information

Supplementary Information Supplementary Sections I–VIII, Figs. 1–7 and Tables I and II.

### Source data

Source Data Fig. 1 Source data for Fig. 1.

Source Data Fig. 2 Source data for Fig. 2.

Source Data Fig. 3 Source data for Fig. 3.

Source Data Fig. 4 Source data for Fig. 4.

## Data Availability

All data that support the plots within this paper and other findings of this study are available from the corresponding authors on reasonable request. Source data are provided with this paper.

## Code availability

The analysis and simulation codes that support the findings of this study are available from the corresponding authors on request.
