# Supplementary material for: Engineering multimode interactions in circuit quantum acoustodynamics
Source: Nat Phys. 2024 Jan 25;20(4):564–70. doi: 10.1038/s41567-023-02377-w (PMC11021184; doi:10.1038/s41567-023-02377-w)
Supplement: Supplementary file 1 — Supplementary Sections I–VIII, Figs. 1–7 and Tables I and II. [file 41567_2023_2377_MOESM1_ESM.pdf]

nature physics

Article

https://doi.org/10.1038/s41567-023-02377-w

Engineering multimode interactions in circuit quantum acoustodynamics

In the format provided by the authors and unedited

CONTENTS

I. Device parameters and measurement setup 1
A. Modulation depth error propagation 1

II. Hamiltonian of a bichromatically driven qubit 1
A. Qubit response 5

III. Modulation depth correction 5

IV. Beam-splitter interaction Hamiltonian 6

V. Drive calibration 9

VI. State tomography 10

VII. Three mode interaction parameters 12
A. Theoretical model 12
B. Fitting routine 12
C. Spurious coupling to additional phonon modes 13

VIII. Bright and dark state hybridization 14

References 16

I. DEVICE PARAMETERS AND MEASUREMENT SETUP

The hybrid device, which we also used for previous studies [1] [2], consists of a transmon qubit and an HBAR resonator, fabricated on separate chips and flip-chip bonded together. We are using up to four high overtone modes of the HBAR resonator: three of them are used in the experiments shown in the main paper and one ancilla mode is used to reset our transmon qubit. In Table I we list the parameters of our device, which we obtained through independent measurements. The sample sits in a 3D aluminum cavity to both shield it from the environment and to serve as a readout mode for the qubit via the dispersive readout commonly used in cQED.
Our microwave cavity is thermalized to the mK stage of a dilution refrigerator and placed inside Mu-Metal shields to shield it from the electromagnetic environment. We generate input signals with a Quantum Machines OPX and upconvert them to GHz frequencies by means of IQ mixers and local oscillators. The input lines are thermalized to each stage, resulting in an effective temperature of the qubit which can be measured by probing its residual thermal population, of approximately 50 mK. Furthermore, we use a SNAIL parametric amplifier [3] to amplify the readout transmitted signals coming from the output port of the aluminum cavity. The amplified readout signal is down-converted using a single-sideband (SSB) mixer and demodulated in the OPX. Fig. 1 shows both the room temperature signal routing and the wiring inside the fridge.

A. Modulation depth error propagation

Our experimental setup allows us to set a modulation depth by choosing the amplitudes $\Omega_{1,2}$ in Eq. (22). The error of this modulation depth is thus a function of the errors of the parameters that go into Eq. (22). The errors of independently measured device parameters are shown in Table I and the error from the calibration of the drive amplitudes is shown in Section V. To compute error bars of Λ', $J_n\left(\lambda'/\Delta_{21}\right)$, and of quantities derived from it, such as the beam-splitter coupling in Eq. (39), we propagate the errors of those system parameters, using standard Gaussian error propagation.

II. HAMILTONIAN OF A BICHROMATICALLY DRIVEN QUBIT

In this section, we present an analytical description of the qubit behavior when driven with the two parametric drives. In particular, we show the emergence of the qubit sidebands in a time-dependent rotating frame and the expression describing the qubit spectroscopy measurements shown in Fig. 1 of the main text.

Variable	Parameter	Value
ω_q	qubit frequency without AC Stark shift	$2\pi \cdot 5.971323\,\mathrm{GHz}{\pm}5\,\mathrm{kHz}$
T_1	qubit relaxation time	$9.5 \pm 0.1\,\mu\mathrm{s}$
T_2^*	qubit coherence time (Ramsey)	$7.2 \pm 0.2\,\mu\mathrm{s}$
T_2^E	qubit coherence time (Echo)	$10.3 \pm 0.3\,\mu\mathrm{s}$
α	qubit anharmonicity	$2\pi \cdot 218 \pm 0.5\,\mathrm{MHz}$
ω_a	resonance frequency of phonon mode a	$2\pi \cdot 5.9236\,\mathrm{GHz}{\pm}2\,\mathrm{kHz}$
ω_b	resonance frequency of phonon mode b	$2\pi \cdot 5.9488\,\mathrm{GHz}{\pm}2\,\mathrm{kHz}$
ω_c	resonance frequency of phonon mode c	$2\pi \cdot 5.9615\,\mathrm{GHz}{\pm}1\,\mathrm{kHz}$
ω_{anc}	resonance frequency of the ancillary mode	$2\pi \cdot 5.9109\,\mathrm{GHz}{\pm}1\,\mathrm{kHz}$
Γ_a	decay rate of phonon mode a	$2\pi \cdot 4.7 \pm 0.1\,\mathrm{kHz}$
Γ_b	decay rate of phonon mode b	$2\pi \cdot 3.3 \pm 0.1\,\mathrm{kHz}$
Γ_c	decay rate of phonon mode c	$2\pi \cdot 2.2 \pm 0.1\,\mathrm{kHz}$
Γ_{anc}	decay rate of the ancillary phonon mode	$2\pi \cdot 1.7 \pm 0.1\,\mathrm{kHz}$
g_m	qubit-phonon coupling	$2\pi \cdot 257 \pm 3\,\mathrm{kHz}$
FSR	HBAR free spectral range	$2\pi \cdot 12.62955\,\mathrm{MHz}{\pm}3\,\mathrm{kHz}$

TABLE I. **List of device parameters.** The errors are the fit uncertainties from the measurements determining the parameters.

We start from the Hamiltonian of a transmon qubit, driven by two parametric drives at frequencies $\omega_{1,2}$ and strength $\Omega_{1,2}$, and a weak probe tone

$$H_{\mathrm{qd}} = \omega_q q^\dagger q - \frac{\alpha}{2} q^{\dagger 2} q^2 + \left(\Omega_1 e^{-i\omega_1 t} q^\dagger + \Omega_2 e^{-i\omega_2 t} q^\dagger + \mathrm{h.c.}\right) + \left(\Omega_p q^\dagger e^{-i\omega t} + \mathrm{h.c.}\right). \tag{1}$$

Here $q, q^\dagger$ are the qubit annihilation and creation operators, α is the qubit anharmonicity and Ω_p, ω are the strength and frequency of the probe tone, respectively. In the rotating frame of the qubit g-e transition (ω_q), the Hamiltonian becomes

$$H'_{\mathrm{qd}} = -\frac{\alpha}{2} q^{\dagger 2} q^2 + \left(\Omega_1 e^{-i\Delta_1 t} q^\dagger + \Omega_2 e^{-i\Delta_2 t} q^\dagger + \mathrm{h.c.}\right) + \left(\Omega_p q^\dagger e^{-i\delta_p t} + \mathrm{h.c.}\right), \tag{2}$$

where $\Delta_{1,2} = \omega_{1,2} - \omega_q$ is the detuning between the drive frequencies and the qubit g-e transition and $\delta_p = \omega - \omega_q$ is the detuning between the probe frequency and the qubit g-e transition. In the following we assume $\delta_p \ll \Delta_{1,2}$. Now we apply a unitary transformation

$$U_{\mathrm{d}} = \exp\left[\xi_1^* e^{i\Delta_1 t} q + \xi_2^* e^{i\Delta_2 t} q - \mathrm{h.c.}\right] \tag{3}$$

to move to the interaction picture, also known as the displaced frame, of these drives, where $\xi_{1,2} = \Omega_{1,2}/\Delta_{1,2}$. This transforms the qubit operator as

$$q' = U_{\mathrm{d}} q U_{\mathrm{d}}^\dagger = \xi_1 e^{-i\Delta_1 t} + \xi_2 e^{-i\Delta_2 t} + q. \tag{4}$$

From now on we will assume that ξ_1 is real-valued and write ξ_2 as $\xi_2 e^{-i\phi}$ with $\xi_2 \in \mathbb{R}$. Under this assumption, ϕ describes the initial phase difference between the two drives. This notation serves to avoid complex drive constants and highlights the effect of an initial phase difference. In our case of two drives, the phase ϕ between them can be gauged away without affecting the dynamics [4]. However, in the presence of an additional phase reference, for example from a third drive, a non-trivial phase difference arises. Tuning this non-trivial phase can lead to interesting effects such as non-reciprocal behavior [5]. Furthermore, we define $\Delta_{21} \equiv \Delta_2 - \Delta_1$, $\Sigma_{21} = \Delta_1 + \Delta_2$, and choose

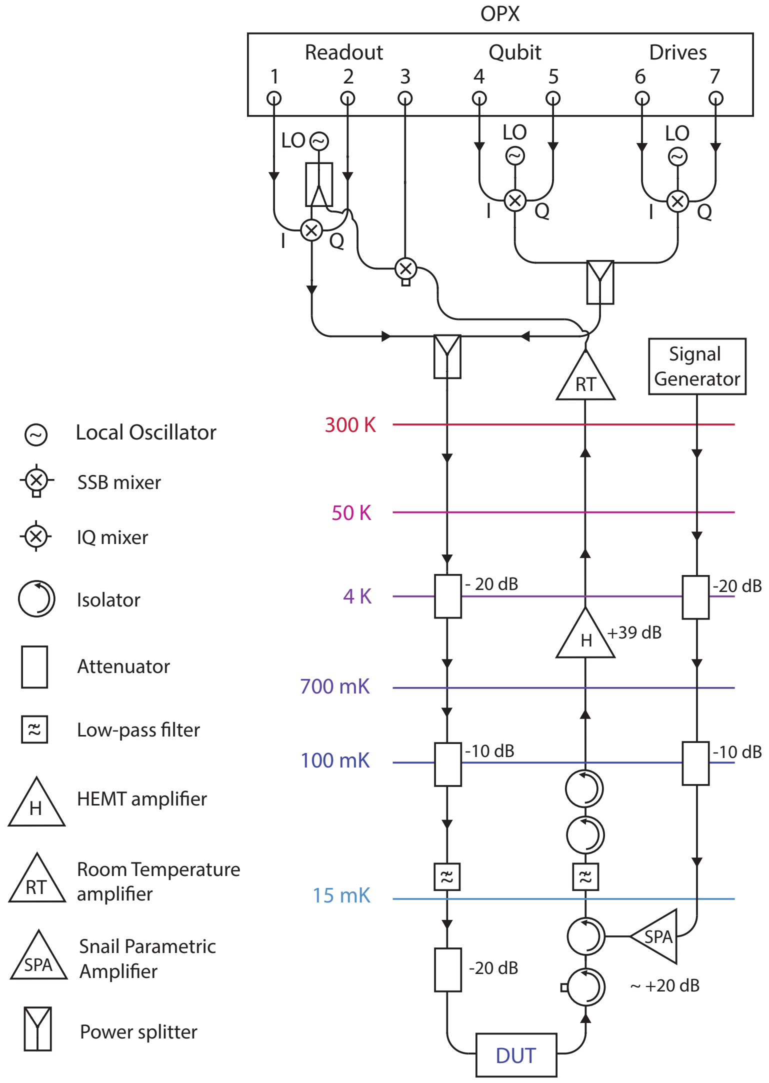

FIG. 1. **Wiring diagram.** In the upper part of the figure, we show the room temperature microwave signal setup in which signals are generated by the OPX and upconverted to GHz frequencies by means of IQ mixers. In the lower part we sketch the cabling inside the fridge, including our Device Under Study (DUT) and the parametric amplifier, both mounted in the mK stage of our dilution fridge.

$\Delta_{21} > 0$. The Hamiltonian $H'_{\rm qd}$ then transforms as

$$
\begin{aligned}
H''_{\rm qd} &= U_d H'_{\rm qd} U_d^\dagger + i\dot{U}_{\rm d} U_{\rm d}^\dagger \qquad (5)\\
&= -\frac{\alpha}{2} {q^\dagger}^2 q^2 \\
&+ \alpha\left(-\xi_1 e^{-i\Delta_1 t} - \xi_2 e^{-i\phi} e^{-i\Delta_2 t}\right) {q^\dagger}^2 q + \text{h.c.} \\
&+ \alpha\left(-2\xi_1^2 - 2\xi_1\xi_2 e^{i\phi} e^{i\Delta_{21} t} - 2\xi_1\xi_2 e^{-i\phi} e^{-i\Delta_{21} t} - 2\xi_2^2\right) q^\dagger q \\
&+ \alpha\left(-\frac{\xi_1^2}{2} e^{-2i\Delta_1 t} - \xi_1\xi_2 e^{-i\phi} e^{-i\Sigma_{21} t} - \frac{\xi_2^2}{2} e^{-2i\phi} e^{-2i\Delta_2 t}\right) {q^\dagger}^2 + \text{h.c.} \\
&+ \alpha\big(- \xi_1^3 e^{-i\Delta_1 t} - \xi_1^2\xi_2 e^{i\phi} e^{-2i\Delta_1 t} e^{i\Delta_2 t} - 2\xi_1^2\xi_2 e^{-i\phi} e^{-i\Delta_2 t} \\
&\quad - 2\xi_1\xi_2^2 e^{-i\Delta_1 t} - \xi_1\xi_2^2 e^{-2i\phi} e^{i\Delta_1 t} e^{-2i\Delta_2 t} - \xi_2^3 e^{-i\phi} e^{-i\Delta_2 t}\big) q^\dagger + \text{h.c.} \\
&+ \left(\Omega_p q^\dagger e^{-i\delta_p t} + \text{h.c.}\right). \qquad (6)
\end{aligned}
$$

Now we will drop all terms oscillating faster than Δ_{21} in a Rotating Wave Approximation (RWA). Note that $\delta_p \simeq \Delta_{21}$, such that we keep the term in the last line of Eq. (6). Since $\Delta_{21} < \Delta_1 < \Delta_2 < \Sigma_{21}$ in our setup, we can drop the terms proportional to the non-diagonal operators ${q^\dagger}^2 q$, ${q^\dagger}^2$, $q^\dagger$ and h.c., except for the probe. Nevertheless, we emphasize that the relation between the different detunings can be modified for a different choice of resonance condition, which could make some of the terms we dropped in this RWA more relevant in other parameter regimes than the ones chosen in this work.

This RWA leads to a Hamiltonian diagonal in the qubit operator, namely

$$
\begin{aligned}
H_{\rm RWA} = &-\frac{\alpha}{2} {q^\dagger}^2 q^2 + \left(-2\alpha\xi_1^2 - 2\alpha\xi_2^2 - 2\alpha\xi_1\xi_2 e^{i\phi} e^{i\Delta_{21} t} - 2\alpha\xi_1\xi_2 e^{-i\phi} e^{-i\Delta_{21} t}\right) q^\dagger q \\
&+ \left(\Omega_p q^\dagger e^{-i\delta_p t} + \text{h.c.}\right) \qquad (7)\\
= &-\frac{\alpha}{2} {q^\dagger}^2 q^2 + \left(\underbrace{-2\alpha\xi_1^2 - 2\alpha\xi_2^2}_{\delta_q^{ss}} \underbrace{-4\alpha\xi_1\xi_2}_{\Lambda} \cos\left(\Delta_{21} t + \phi\right) \right) q^\dagger q + \left(\Omega_p q^\dagger e^{-i\delta_p t} + \text{h.c.}\right). \qquad (8)
\end{aligned}
$$

In Eq. (8) the expression in large brackets represents the Stark shift of the qubit due to the presence of the parametric drives, also shown as Eq. (2) in the main text. This term has a time-independent part $\left(\delta_q^{ss}\right)$ and a time-dependent part (term with Λ as prefactor), the former acting as a frequency shift of the qubit and the latter as a modulation of the qubit frequency. The modulation has a modulation frequency Δ_{21} and modulation depth Λ/Δ_{21}. We move our qubit rotating frame to that of the Stark shifted qubit, $q \to q e^{-i\delta_q^{ss} t}$, which removes the constant frequency shift δ_q^{ss} and modifies the detuning of the probe tone to $\tilde{\delta}_p = \delta_p - \delta_q^{ss}$. Note that δ_q^{ss} acquires a correction in the parameter regime we work in, which is shown in Section V. Entering this rotating frame also has an effect on coupling terms, which we will introduce later when we treat the phonon modes. In this frame the qubit Hamiltonian is

$$
H'_{\rm RWA} = -\frac{\alpha}{2} {q^\dagger}^2 q^2 - 4\alpha\xi_1\xi_2 \cos\left(\Delta_{21} t + \phi\right) q^\dagger q + \left(\Omega_p q^\dagger e^{-i\tilde{\delta}_p t} + \text{h.c.}\right) . \qquad (9)
$$

Next we move into the time-dependent rotating frame of the frequency modulation by applying the transformation

$$
U_{\rm JA} = \exp\left[i\frac{\Lambda}{\Delta_{21}} \sin\left(\Delta_{21} t + \phi\right) q^\dagger q\right]. \qquad (10)
$$

This removes the time-dependent qubit frequency term in Eq. (9) since

$$
i\dot{U}_{\rm JA} U_{\rm JA}^\dagger = -\Lambda \cos\left(\Delta_{21} t + \phi\right) q^\dagger q \qquad (11)
$$

and transforms the qubit operator according to the Jacobi-Anger expansion as

$$
q_{\rm JA} = q \exp\left[-i\frac{\Lambda}{\Delta_{21}} \sin\left(\Delta_{21} t + \phi\right)\right] = q \sum_{n=-\infty}^{\infty} J_n\left(\frac{\Lambda}{\Delta_{21}}\right) e^{-in(\Delta_{21} t + \phi)}, \qquad (12)
$$

where J_n is the Bessel function of the first kind and n refers to the sideband number.

Since this frame transformation leads to a time-dependent phase in the qubit operator, it does not affect the diagonal terms in H_{RWA}, but it does affect the probe term as we can see in the transformed Hamiltonian

$$H_{\mathrm{JA}} = -\frac{\alpha}{2} q^{\dagger 2} q^2 + \left(\Omega_p q^\dagger \sum_{n=-\infty}^{\infty} J_n \left(\frac{\Lambda}{\Delta_{21}} \right) e^{in(\Delta_{21} t + \phi)} e^{-i\tilde{\delta}_p t} + \text{h.c.} \right). \tag{13}$$

By noting that Ω_p is small compared to Δ_{21} we can perform an additional RWA to keep only time-independent terms. Thus, we keep only the terms which satisfy the condition $n\Delta_{21} = \tilde{\delta}_p$, leading to the interpretation that the probe only affects the qubit when it is detuned from the Stark shifted g-e transition by integer multiples of Δ_{21}. An experimental confirmation of this can be seen through the appearance of multiple qubit "sidebands" during qubit spectroscopy (see Fig. 1**b** of the main paper).

A. Qubit response

To model the spectroscopic response of our transmon qubit when driven by two tones and probed by a third weak tone we make use of the Bloch equations in steady state, and write the excited state population [6] as

$$P_e = \frac{1}{2} \sum_{n=-\infty}^{\infty} \frac{\Omega_n^2 T_1 T_2^*}{1 + (T_2^* \Delta)^2 + \Omega_n^2 T_1 T_2^*}. \tag{14}$$

Here T_1 is the energy relaxation time, T_2^* is the Ramsey decoherence time and $\Delta = \omega - \omega_q - n\Delta_{21}$ is the detuning between the probe tone and the generated qubit sidebands. Furthermore, the relative probe strength can be expressed as

$$\Omega_n = J_n \left(\frac{\Lambda}{\Delta_{21}} \right) \Omega_p. \tag{15}$$

We use Eq. (14) to model the data shown in Fig. 1 of the main paper.

III. MODULATION DEPTH CORRECTION

In Section II we saw how going into the interaction picture of the two parametric drives leads to a time-dependent Stark shift on the qubit. The resulting time-dependent term acts like a frequency modulation, creating sidebands whose amplitude is proportional to the Bessel function of the first kind. The argument of the Bessel functions is the modulation depth Λ/Δ_{21}, where $\Lambda = -4\alpha\xi_1\xi_2$ is the prefactor of the cosine modulation of the qubit frequency. However, when taking into account higher energy levels of the qubit, its time-dependent response to the parametric drives becomes more complex, in a similar fashion to how the time-independent Stark shift depends on the qubit anharmonicity in Eq. (44). This is the result of ignoring the Kerr-term in Eq. (28). Here, we use time-independent perturbation theory to derive a correction to the modulation depth due to this effect. The calculation of the correction is straightforward for a single mode where we can enter a rotating frame at the drive frequency, thereby eliminating the explicit time dependence of the drive term as done for example in [4]. However, this is not obvious for our Hamiltonian which includes two drives. To mitigate this, we model our drives as harmonic modes with resonance frequencies $\omega_{1,2}$. These are described by the operators $d_{1,2}^{(\dagger)}$ and are coupled to the qubit with coupling strengths $g_{1,2}$. The Hamiltonian of the qubit and drives then reads

$$H = H_0 + \lambda V \ , \tag{16}$$

where

$$H_0 = \omega_q q^\dagger q - \frac{\alpha}{2} q^{\dagger 2} q^2 + \omega_1 d_1^\dagger d_1 + \omega_2 d_2^\dagger d_2 \tag{17}$$

$$V = g_1 d_1^\dagger q + g_2 d_2^\dagger q + \text{h.c.} \tag{18}$$

Keeping explicit field terms $d_{1,2}^\dagger d_{1,2}$ eliminates the explicit time dependence. We now write the joint state of qubit and drives as $|nlm\rangle$, where the indices n, l, m are the photon numbers in qubit (n) and the two drive modes (l, m). By following standard perturbation theory we find the unperturbed energy levels $E_{nlm}^{(0)}$

$$H_0|nlm\rangle = \Big(\omega_q n - \frac{\alpha}{2} n(n-1) + \omega_1 l + \omega_2 m\Big)\,|nlm\rangle \equiv E^{(0)}_{nlm}|nlm\rangle \ , \tag{19}$$

with the first order correction

$$E^{(1)}_{nlm} = \langle nlm|V|nlm\rangle = 0 \ , \tag{20}$$

and the second order correction

$$E^{(2)}_{nlm} = \sum_{k\neq n} \frac{|\langle kst|V|nlm\rangle|^2}{E^{(0)}_{nlm} - E^{(0)}_{kst}} \ . \tag{21}$$

Note that this second order correction to the energy levels contains many terms which we handle in Wolfram Mathematica and do not reproduce here. Nevertheless, with this long but analytically tractable expression for $E^{(2)}_n$ in hand, we compute the correction to the transition between neighboring levels $E^{(2)}_n - E^{(2)}_{n-1}$. After evaluating Eq. (21) for our Hamiltonian, we identify the "coupling strengths" as being given by the drive amplitudes scaled by the square root of the photon number in each drive $g_{1,2} \to \Omega_{1,2}/\sqrt{l,m}$. This allows us to eliminate the drive occupations l, m from the result. Next, we identify terms proportional to $\Omega_1\Omega_2^\star$ + h.c. as those contributing to the frequency modulation and terms proportional to $|\Omega_{1,2}|^2$ as those contributing to the Stark shift. Focusing solely on the modulation depth correction we finally arrive at the correction for the lowest qubit transition

$$E^{(2)}_1 - E^{(2)}_0 = -2\alpha\Omega_1\Omega_2 \left(\frac{1}{\Delta_1(\Delta_1+\alpha)} + \frac{1}{\Delta_2(\Delta_2+\alpha)}\right) \equiv \Lambda' \ , \tag{22}$$

where $\Delta_{1,2} = \omega_{1,2} - \omega_q$ as in the previous sections. In the figures in the main text, we use this corrected prefactor of the qubit frequency modulation to translate the drive amplitudes $\Omega_{1,2}$ into a corrected modulation depth Λ'/Δ_{21}. Furthermore, note that we take into account the Stark shift correction separately in Section V.

IV. BEAM-SPLITTER INTERACTION HAMILTONIAN

In Section II we have seen what happens to the Hamiltonian of a bichromatically driven qubit when probed by a weak probe tone. In this section, we will study the effect of the driven qubit on a finite number of harmonic oscillators, for example two phonon modes a and b.
The initial Hamiltonian in the lab frame and without the probe tone is given by

$$H_{\rm JC} = \omega_q q^\dagger q - \frac{\alpha}{2} {q^\dagger}^2 q^2 + \big(\Omega_1 e^{-i\omega_1 t} q^\dagger + \Omega_2 e^{-i\omega_2 t - i\phi} q^\dagger + \text{h.c.}\big) + \omega_a a^\dagger a + \omega_b b^\dagger b + \big(g_a a^\dagger q + g_b b^\dagger q + \text{h.c.}\big) \tag{23}$$

Now we apply the same transformations as in Section II and, in addition, also move the phonons to a frame rotating with their resonance frequencies $\omega_{a,b}$. Right before we enter the time-dependent rotating frame with the transformation $U_{\rm JA}$ in Eq. (10), the Hamiltonian is

$$\begin{aligned} H^{\rm m}_{\rm RWA} = &- \frac{\alpha}{2} {q^\dagger}^2 q^2 \underbrace{-4\alpha\xi_1\xi_2}_{\Lambda} \cos(\Delta_{21} t + \phi) q^\dagger q \\ &+ \Big(g_a a^\dagger q e^{i\tilde{\Delta}_a t} + g_b b^\dagger q e^{i\tilde{\Delta}_b t} + \text{h.c.}\Big) \\ &+ \big(\big(g_a a^\dagger e^{i\Delta_a t} + g_b b^\dagger e^{i\Delta_b t}\big)\big(\xi_1 e^{-i\Delta_1 t} + \xi_2 e^{-i\Delta_2 t - i\phi}\big) + \text{h.c.}\big) \ , \end{aligned} \tag{24}$$

with $\tilde{\Delta}_{a,b} = \omega_{a,b} - (\omega_q + \delta^{ss}_q)$ and the modulation depth Λ as in Section II.

The second line of Eq. (24) describes the off-resonant coupling between qubit and phonon modes and the third line represents an effective drive on the phonon modes mediated by the qubit. Note that the strength of the effective drive depends on the detuning $\Delta_{a,b}$ between the bare qubit and the phonon frequencies as it is the result of the transformation $U_{\rm d}$ in Eq. (3). Its resonance condition depends on the phonon-drive detuning $\Delta_{a,b} - \Delta_{1,2}$, which is large for the parametric drives considered here, such that we drop the effective drive terms.

Now applying $U_{\rm JA}$ and also using the shorthand $J_n(\frac{\Lambda}{\Delta_{21}}) \equiv J_n$ for better readability, we get

$$H^{\rm m}_{\rm JA} = \underbrace{-\frac{\alpha}{2} {q^\dagger}^2 q^2}_{H_{\rm Kerr}} + \underbrace{\left(g_a a^\dagger q \sum_n J_n e^{i(\tilde{\Delta}_a - n\Delta_{21})t} e^{-in\phi} + g_b b^\dagger q \sum_k J_k e^{i(\tilde{\Delta}_b - k\Delta_{21})t} e^{-ik\phi} + \text{h.c.}\right)}_{H_{\rm sideband\ coupling} = V}, \tag{25}$$

where both sums run from $-\infty$ to ∞. Note that for better readability, we use the indices n and k to represent the sideband number for the $a^\dagger q$ term and the $b^\dagger q$ term.

From the second term of Eq. (25), we see that the qubit sidebands (which are generated by the frequency modulation arising from the two drives) couple individually to the phonon modes. This can be used to activate and control a coupling between the qubit and a detuned phonon mode m with a coupling $g_m J_n$ by varying Λ and Δ_{21} [7, 8].

Moreover, the second line of Eq. (25) can be understood as an off-resonant coupling between the qubit and the two phonon modes (a and b) through each sideband (J_n and J_k). The coupling via the $n(k)^{th}$ sideband comes with a phase $\tilde{\Delta}_{a(b)} - n(k)\Delta_{21}$, such that most of the infinite sum terms are fast oscillating. The sideband closest to the respective phonon mode is detuned only by about 1 MHz in our experiment, such that it leaves a small residual JC-interaction between qubit and phonon.

We now move into an interaction picture of the coupling between the qubit and the phonon modes through the different sidebands via the transformation

$$U_c = \exp\left[\sum_n \frac{g_a J_n}{\tilde{\Delta}_a - n\Delta_{21}} a^\dagger q e^{i(\tilde{\Delta}_a - n\Delta_{21})t} e^{-in\phi} + \sum_k \frac{g_b J_k}{\tilde{\Delta}_b - k\Delta_{21}} b^\dagger q e^{i(\tilde{\Delta}_b - k\Delta_{21})t} e^{-ik\phi} - \text{h.c.}\right] \equiv e^S. \tag{26}$$

Note that this is analogous to the usual Schrieffer-Wolff transformation used in the dispersive regime of a qubit and resonator coupled through the Jaynes-Cummings interaction. The transformed Hamiltonian is

$$\begin{aligned} H_c &= U_c H_{\mathrm{JA}}^{\mathrm{m}} U_c^\dagger + i\dot{U}_{\mathrm{c}} U_{\mathrm{c}}^\dagger & (27)\\ &= U_c H_{\mathrm{Kerr}} U_c^\dagger + U_c V U_c^\dagger + i\frac{\partial}{\partial t}\left(e^S\right) e^{-S} & (28) \end{aligned}$$

$U_c H_{\mathrm{Kerr}} U_c^\dagger$ yields terms comprised of a total of four phonon (a, b) and qubit (q) operators with appropriate prefactors. The photon number dependence of the qubit transition frequency described by H_{Kerr} leads to a modification of the effective drive strengths and of the modulation depth, which we describe in the following section [9]. This modification also applies to the effective beam-splitter coupling rates, but we neglect this effect in our analysis as it is negligible if $\Delta_{a,b} \ll \alpha$. Note that this is not the case for some previous works [10], where the correction would have to be taken into account to correctly predict the coupling rates and frequency shifts. Furthermore, H_{Kerr} creates combinations of phonon and qubit operators with drive-related terms arising from the transformation in Eq. (5). These forth order terms become relevant when their respective resonance condition is met and their amplitudes are large enough. Their effects are both interesting for further studies and troublesome when overshadowing the physics we want to highlight in this work. Even though for the experiments presented here we avoided hitting these resonances, they might pose a challenge when scaling up to a larger number of simultaneously driven interactions.

Our main focus is the interaction term V and how the transformation U_c reveals the beam-splitter interaction between the phonon modes. To that end, we use the Baker-Campbell-Hausdorff formula to explicitly execute the above transformation. Noting that $i\partial S/\partial t = -V$ and expanding the derivative of e^S as a Taylor series, we can write the

effect of V under the coupling interaction picture U_c as

$$U_c V U_c^\dagger + i\frac{\partial}{\partial t}\left(e^S\right)e^{-S} \tag{29}$$

$$=\sum_{j=0}^{\infty}\frac{1}{j!}\left(\mathrm{ad}_S\right)^j V + i\sum_{j=0}^{\infty}\frac{1}{(j+1)!}\left(\mathrm{ad}_S\right)^j\frac{\partial S}{\partial t} \tag{30}$$

$$=V+[S,V]+i\frac{\partial S}{\partial t}+i\frac{1}{2}\left[S,\frac{\partial S}{\partial t}\right]+\mathcal{O}\left(g_{a,b}^3/\Delta_{a,b}^3\right) \tag{31}$$

$$=V+[S,V]-V-\frac{1}{2}[S,V]+\mathcal{O}\left(g_{a,b}^3/\Delta_{a,b}^3\right) \tag{32}$$

$$=\frac{1}{2}[S,V] \tag{33}$$

$$=\underbrace{\sum_{n,k}\frac{g_a^2 J_n J_k}{\tilde{\Delta}_a-n\Delta_{21}}\cos\left[(k-n)\Delta_{21}t+(k-n)\phi\right]a^\dagger a}_{\delta_a} \tag{34}$$

$$+\underbrace{\sum_{n,k}\frac{g_b^2 J_n J_k}{\tilde{\Delta}_b-k\Delta_{21}}\cos\left[(k-n)\Delta_{21}t+(k-n)\phi\right]b^\dagger b}_{\delta_b} \tag{35}$$

$$-\underbrace{\sum_{n,k}\left\{\frac{g_a^2 J_n J_k}{\tilde{\Delta}_a-n\Delta_{21}}+\frac{g_b^2 J_n J_k}{\tilde{\Delta}_b-k\Delta_{21}}\right\}\cos\left[(k-n)\Delta_{21}t+(k-n)\phi\right]q^\dagger q}_{\delta_q} \tag{36}$$

$$+\underbrace{\sum_{n,k}\frac{1}{2}\left\{\frac{g_a g_b J_n J_k}{\tilde{\Delta}_b-k\Delta_{21}}+\frac{g_a g_b J_n J_k}{\tilde{\Delta}_a-n\Delta_{21}}\right\}e^{i(k-n)\Delta_{21}t}e^{i(\Delta_a-\Delta_b)t}e^{i(k-n)\phi}}_{g_{\mathrm{BS}}}a^\dagger b+\mathrm{h.c.} \tag{37}$$

where $\mathrm{ad_S}\,\bullet\equiv[S,\ \bullet]$ in Eq. (30) denotes the adjunct action. A thorough derivation of Eqs. (29) to (33) can be found in the appendix of Ref. [11]. Eq. (34) and Eq. (35) contain phonon frequency shifts $\delta_{a,b}$ due to the presence of the qubit and its sidebands, with Eq. (36) containing an equal, but opposite shift of the qubit frequency. As discussed in Ref. [12], these phonon frequency shifts are predominantly due to the normal mode splitting of qubit and phonon modes when approaching an avoided crossing. The qubit frequency shifts by the same amount in the opposite direction, evident from Eq. (36). Finally, Eq. (37) unveils the phonon-phonon beam-splitter coupling. We can formally eliminate the phonon frequency shifts by entering a rotating frame for the phonon modes which cancels the shifts and adds a corresponding phase to the operators a and b. This modifies the resonance condition of Eq. (37) to $(n-k)\Delta_{21}=\Delta_a-\Delta_b+\delta_a-\delta_b$, similar to how the qubit Stark shift modified the qubit-phonon detunings from $\Delta_{a,b}$ to $\tilde{\Delta}_{a,b}$ In other words, the difference between the two drive frequencies now has to match the difference between the shifted phonon frequencies. To realize this modified resonance condition in the experiment we need to adapt Δ_{21}, which slightly changes the prefactor in Eq. (37) as we change the denominator. However, we can safely assume that $|\tilde{\Delta}_b-k\Delta_{21}|\gg|\delta_{a,b}|$, so this change will be small.

Even after eliminating the phonon frequency shifts, we are still left with an unwieldy term for the beam-splitter coupling, which contains two infinite sums and multiple phase factors. Thus, to simplify the Hamiltonian further, we make use of the four-wave mixing resonance condition, noting that the phase of the term should remain constant in time. This allows us to write a condition for k in terms of n to eliminate the sum over k since both the phonon-phonon detuning $\Delta_a-\Delta_b$ and the drive frequency difference Δ_{21} are given by the experiment. In the main text, we are using in particular the phonon frequency shifts as well as the coupling terms that arise between neighboring phonon modes when $n=k+1$ and next-to-neighboring phonon modes when $n=k+2$. These three terms are simplifications of Eq. (34) and (37), using $\phi=0$ and $(n-k)\Delta_{21}=\Delta_a-\Delta_b$, leading to Eq. (3), (4), and (5) of the main text, which we repeat here for a phonon mode m, detuned from the qubit by Δ_m

$$\delta_m=g_m^2\sum_n\frac{J_n^2}{\Delta_m-n\Delta_{21}}, \tag{38}$$

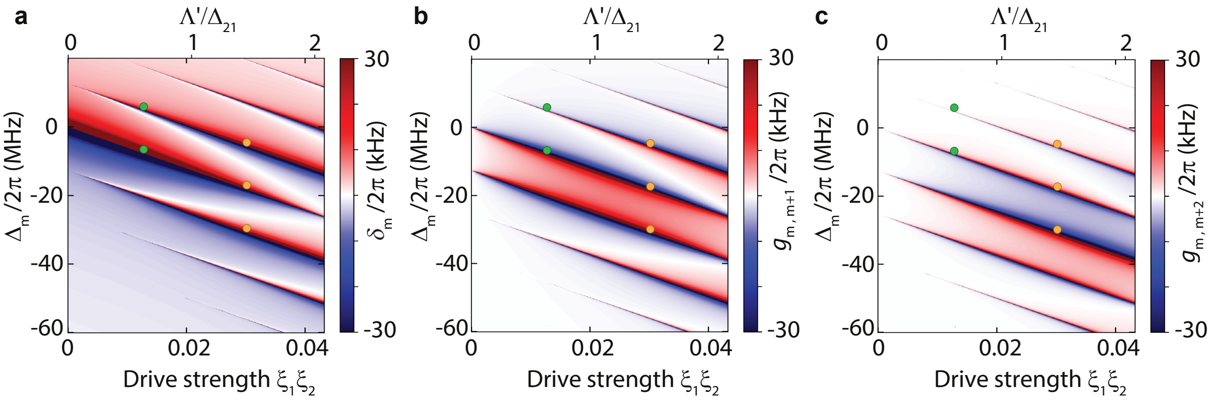

FIG. 2. **Analytical result for phonon frequency shift and beam-splitter coupling.** **a** Frequency shift of a phonon mode m detuned from the qubit by $\Delta_m = \omega_m - \omega_q$ for a drive power $\xi_1\xi_2$. Green (orange) circles indicate the parameters of the 2 (3) phonon modes used in the experiments presented in Fig. 2 (3) of the main text. **b** Beam-splitter coupling of the same mode to its first higher frequency neighbor $m+1$ at $\Delta_m + \Delta_{21}$. **c** Beam-splitter coupling from mode m to its second higher frequency neighbor $m+2$ at $\Delta_m + 2\Delta_{21}$.

$$g_{\rm BS}^{m,m+1} = g_m g_{m,m+1} \sum_n \frac{J_n J_{n+1}}{\Delta_m - n\Delta_{21}}, \tag{39}$$

$$g_{\rm BS}^{m,m+2} = g_m g_{m,m+2} \sum_n \frac{J_n J_{n+2}}{\Delta_m - n\Delta_{21}}. \tag{40}$$

To illustrate how Eq. (38), (39), and (40) behave for various drive strengths and phonon-qubit detunings, we plot them in Fig. 2. The diagonal lines correspond to qubit frequencies, where the denominator $\Delta_m - n\Delta_{21}$ diverges. Note that we omitted the Stark shift correction of Δ_m, so that the effect of the Stark shift can be seen in the downward slope of the features in Fig. 4. The green (orange) circles indicate the parameters of the phonon modes used in the experiments presented in Fig. 2 (3) of the main text.

Lastly, we note that to first order in the BCH-expansion, the qubit and phonon operators transform as

$$U_{\rm c} q U_{\rm c}^\dagger = q + \sum_n \frac{g_a J_n}{\tilde\Delta_a - n\Delta_{21}} a e^{-i(\tilde\Delta_a - n\Delta_{21})t} e^{in\phi} + \sum_k \frac{g_b}{\tilde\Delta_b - k\Delta_{21}} b e^{-i(\tilde\Delta_b - k\Delta_{21})t} e^{ik\phi} \tag{41}$$

$$U_{\rm c} a U_{\rm c}^\dagger = a - \sum_n \frac{g_a J_n}{\tilde\Delta_a - n\Delta_{21}} q e^{i(\tilde\Delta_a - n\Delta_{21})t} e^{-in\phi} \tag{42}$$

$$U_{\rm c} b U_{\rm c}^\dagger = b - \sum_k \frac{g_b J_k}{\tilde\Delta_b - k\Delta_{21}} q e^{i(\tilde\Delta_b - k\Delta_{21})t} e^{-ik\phi} \tag{43}$$

These transformations provide a more intuitive understanding of the possible combinations of phonon and qubit operators that can arise when transforming $H_{\rm Kerr}$ and $H_{\rm drive}$. Essentially, the qubit partially hybridizes with all phonon modes through the sideband mediated interaction, allowing us to replace the qubit and phonon operators with the respective hybridized operators, similar to what is done in a standard Schrieffer-Wolff transformation.

V. DRIVE CALIBRATION

We want to calibrate the ratio $\eta_{1,2}$ between the effective drive amplitudes $\Omega_{1,2}$ reaching the qubit and the unitless amplitudes $\Omega_{1,2}^{\rm DAC}$ we set for the digitally created waveforms. To that end we perform a spectroscopy measurement where for varying values of $\Omega_{1,2}^{\rm DAC}$, we extract the qubit's Stark-shifted frequency through a Lorentzian fit. We perform this experiment for one drive at a time while the other one is turned off. Since the drives are spaced by $\Delta_{21} = 2\pi \cdot 12.62955$ MHz ($\omega_2 > \omega_1$) the power dependent frequency shift of the qubit will be different for each of the

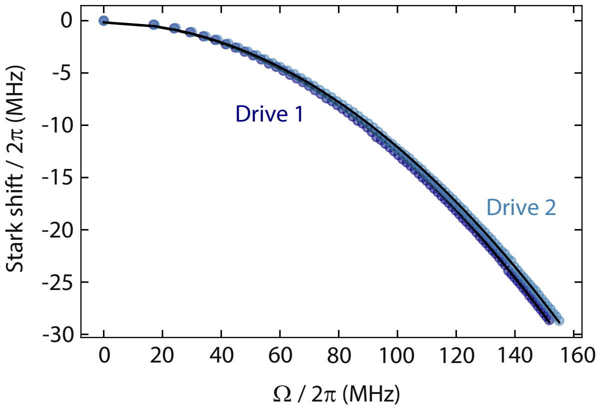

FIG. 3. **Stark shift versus drive amplitude.** The extracted qubit frequency is plotted versus drive amplitude for both drive 1 (dark blue) and drive 2 (light blue). Note that in this measurement and throughout the work presented in this paper the drive frequencies are $\omega_1 = \omega_\mathrm{q} + 2\pi \cdot 492.552\,\mathrm{MHz}$ and $\omega_2 = \omega_\mathrm{q} + 2\pi \cdot 505.182\,\mathrm{MHz}$, respectively. The fit curves performed with Eq. (44) are plotted as black lines.

tones. The frequency shift is given by an expression analogous to that for the dispersive shift between a transmon qubit and a 3D cavity found in Ref. [9]:

$$\delta_q^{ss'} = -2|\Omega_{1,2}|^2 \frac{\alpha}{\Delta_{1,2}(\alpha + \Delta_{1,2})} = -2|\eta_{1,2}\Omega_{1,2}^{\mathrm{DAC}}|^2 \frac{\alpha}{\Delta_{1,2}(\alpha + \Delta_{1,2})}. \tag{44}$$

In the regime where $\alpha \ll \Delta_{1,2}$, Eq. (44) reduces to δ_q^{ss} in Eq. (8).

In Supplementary Fig. 3, we show the extracted Stark shifts versus drive amplitudes as well as the fit to Eq. (44). Note that since the qubit anharmonicity is already known from independent measurements, the conversion factor is the only fitting parameter. From the fits we extract $\eta_1 = 2\pi \cdot 256 \pm 0.17\,\mathrm{MHz}$ and $\eta_2 = 2\pi \cdot 262 \pm 0.17\,\mathrm{MHz}$, where the error is the standard deviation returned by the fit. These will be used later on to estimate the relative drive amplitudes $\xi_{1,2}$ when the qubit is bichromatically driven. In the experiments presented in the main text, we use $\Omega_1^{\mathrm{DAC}} = \Omega_2^{\mathrm{DAC}}$, resulting in $\xi_1 \approx \xi_2$.

VI. STATE TOMOGRAPHY

In this section, we outline the measurements and data analysis performed for the qubit state tomography shown in Fig. 2**e** of the main text. Following the process described in Ref. [13], we measure the different quadratures of the state in the two phonon modes and reconstruct a physical density matrix. As this procedure is well established in the field of cQED, we focus mostly on the determination of our measurement fidelity and cover

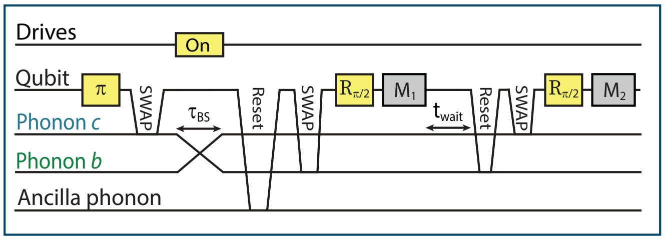

FIG. 4. **Beam-splitter and tomography sequence.** We start by loading an excitation into phonon mode c. Then, we activate the beam-splitter interaction for the duration of a $\sqrt{\mathrm{iSWAP}}$-gate and remove any residual qubit population by performing a SWAP with an ancilla mode. Afterwards we swap the state of phonon mode b into the qubit, perform a $\pi/2$-rotation and measure the qubit state. After this operation we wait for $t_\mathrm{wait} = 6\,\mu\mathrm{s}$ and reset the qubit with the already emptied phonon mode b. Finally, we swap the state of phonon c into the qubit, perform another $\pi/2$-rotation, and measure the qubit state again.

Step	Calibration measurement	$\|g\rangle$-Fidelity	$\|e\rangle$-fidelity
SWAP phonon b-qubit	Vacuum Rabi oscillation between qubit and phonon b	100%	88.3(3)%
SWAP phonon c-qubit	Vacuum Rabi oscillation between qubit and phonon c	100%	90.4(3)%
6us wait time	T_1-measurement phonon c	100%	92.1(2)%
Qubit state assignment	Single-shot calibration via qubit amplitude Rabi	93.4(2)%	78.8(2)%

TABLE II. **Fidelities associated with individual measurement steps.** We calibrate the fidelities of the SWAP-interaction between a phonon mode and the qubit by exciting the qubit and moving it on resonance with the phonon mode. The decaying oscillation lets us fit for the fidelity per SWAP. The fidelity associated with having phonon c idle while we are measuring phonon b and waiting for the cavity to thermalize is given by the decay time of phonon c and the wait time. We quantify the single-shot fidelity of the qubit readout from the contrast of an amplitude Rabi measurement.

the rest only in broad strokes. At the end we compute the degree of entanglement using various entanglement metrics.

To treat our two phonon modes like two-level systems, we rely on the assumption that the state is mostly contained within their two lowest energy levels. Note that we take into account any leakage into higher Fock levels in the final fidelity error. Under this assumption and in order to measure the joint operators of the two-phonon state after a certain sequence, we repeat the following for each phonon mode:

1. Swap the state of interest from the phonon mode to the qubit via a SWAP operation
2. Perform a rotation to measure a desired quadrature ($\mathcal{R}_Y^{-\pi/2}$ for X, $\mathcal{R}_X^{\pi/2}$ for Y, and $\mathcal{I}$ for Z)
3. Dispersive readout of the qubit through a microwave resonator

Between two qubit measurements in each sequence, we wait for a time $t_{\text{wait}} = 6\,\mu s$ to allow the readout resonator to return to its ground state. The full sequence, including the state preparation and beam-splitter interaction, is shown in Supplementary Fig. 4. Together, the two subsequent measurements allow us to measure all two-qubit operators AB, where $A, B \in \{I, X, Y, Z\}$. We perform 5000 single-shot measurements for each measurement operator, and their average yields the measured probabilities $\mathbf{P_M} = \left(P_{AB}^{00},\ P_{AB}^{01},\ P_{AB}^{10},\ P_{AB}^{11}\right)$. We then convert the measured probabilities into expectation values $\mathbf{m_M} = (\langle II\rangle,\ \langle IB\rangle,\ \langle AI\rangle,\ \langle AB\rangle)$ by using the inverse fidelity matrix $[\beta_a\beta_b]^{-1}$ as follows

$$\mathbf{m_M} = [\beta_a\beta_b]^{-1}\mathbf{P_M}. \tag{45}$$

By including the fidelities of the different steps of our measurement routine into the fidelity matrices $\beta_{1,2}$, we can exclude the associated infidelities from our measured density matrix ρ_M. The measured density matrix is then calculated from the measurement operators AB and the corrected expectation values $\langle AB\rangle$ via

$$\rho_M = \sum_{A,B} \frac{1}{4}\langle AB\rangle AB. \tag{46}$$

To determine the single phonon fidelity matrices $\beta_{a,b}$, we independently measure the fidelities associated with the phonon-qubit SWAP operations, the waiting time after the first measurement, and the qubit state assignment. Table II shows the independent measurement used for calibration of each step, as well as the fidelities associated with starting in a $|g\rangle$ or an $|e\rangle$ state in the phonon. With this, we arrive at g[e]-state measurement fidelities of 93.4(2)% [65.6(6)%] for phonon a and 93.4(2)% [69.5(4)%] for phonon b.

We emphasize that accounting for the infidelities of our measurement in this way does not guarantee a physical density matrix. Therefore, we use a Maximum Likelihood Estimation (MLE) to fit the parameters of the Cholesky decomposition of a physical density matrix ρ_T to the measured density matrix ρ_M. This allows to arrive at the density matrix ρ_{MLE} presented in Fig. 2**e** of the main paper [13]. Finally, to quantify the degree of entanglement of the state we created, we compute the overlap of our reconstructed state with an anti-symmetric Bell state, $\mathcal{F}_{\text{Bell}} = \text{Tr}\left\{\sqrt{\sqrt{\rho_T}\rho_{\text{Bell}}\sqrt{\rho_T}}\right\}^2 = 0.69 \pm 0.01$. For the error on on the overlap, we compute the sampling error by Monte-Carlo propagation of the sampling errors on the measured probabilities $\mathbf{P_M}$, using 5000 sets of randomly probabilities from within $\mathbf{P_M} \pm \sigma_m/\sqrt{n}$, where σ_m is the standard deviation of the projective measurements and $n = 5000$ is the number of shots per projection operator. We then propagate the errors of the infidelity shown in Table II and the sampling error to reach the errors given above.

VII. THREE MODE INTERACTION PARAMETERS

In this section we show how we extract the effective coupling strengths and phonon frequency shifts from the phonon population data, an example of which is shown in Fig. 3**b** and **c** of the main text. We perform a 2D fitting routine based on the following model.

A. Theoretical model

The model we use in our fitting routine is based on the equations of motion (EOMs) of five coupled harmonic oscillators. We take into account the beam-splitter coupling between all mode pairs as well as their individual frequency shifts and decay rates. We keep the naming convention for the three modes presented in the main text, a, b, and c, and consider one additional mode on each side, i.e. we consider, from low to high frequency, the modes d, a, b, c, and e. We assume here that residual direct JC interactions with the qubit are off-resonant and can be neglected. In practice this is only approximately true, but smoothing the data removes the resulting fast oscillating, low amplitude effects and leaves only those relevant to the phonon-phonon interaction. To avoid long simulation times when numerically solving the EOMs, we set the frequency of mode d to $\omega_d = 0$. In addition, we move to a rotating frame in which the effective beam-splitter couplings are time-independent, i.e. $U_{\mathrm{RF}} = \exp\{-i(\Delta a^\dagger a + 2\Delta b^\dagger b + 3\Delta c^\dagger c + 4\Delta e^\dagger e)t\}$, where for this section $\Delta \equiv \mathrm{FSR}$. We vary $\Delta_{21} - \Delta \equiv \delta$ in our experiment to probe the resonance condition of different phonon-phonon beam-splitter interactions. In addition, each phonon experiences a frequency shift δ_m due to the normal mode splitting with the qubit (main text Eq. (3)). Therefore, the Hamiltonian in the rotating frame is

$$\begin{aligned} H_{\mathrm{RF}} &= (\delta_a - \delta_d + \delta)a^\dagger a + (\delta_b - \delta_d + 2\delta)b^\dagger b + (\delta_c - \delta_d + 3\delta)c^\dagger c + (\delta_e - \delta_d + 4\delta)e^\dagger e \\ &\quad + \sum_{m,k\in\{d,a,b,c,e\}} g_{mk} m^\dagger k + \mathrm{h.c.} \end{aligned} \tag{47}$$

The first line of Eq. (47) contains the mode frequency terms and the second line contains the coupling terms between all five modes with effective beam-splitter couplings g_{mk}. Here, we consider $g_{mk} \in \mathcal{R}$ following Eq. (23). Writing $\delta_k - \delta_m \equiv \delta_{mk}$, the EOMs are given by

$$\begin{pmatrix} \dot{d}(t) \\ \dot{a}(t) \\ \dot{b}(t) \\ \dot{c}(t) \\ \dot{e}(t) \end{pmatrix} = -i \begin{pmatrix} -i\Gamma_d & g_{da} & g_{db} & g_{dc} & g_{de} \\ g_{da} & \delta_{da} + \delta - i\Gamma_a & g_{ab} & g_{ac} & g_{ae} \\ g_{db} & g_{ab} & \delta_{db} + 2\delta - i\Gamma_b & g_{bc} & g_{be} \\ g_{dc} & g_{ac} & g_{cb} & \delta_{dc} + 3\delta - i\Gamma_c & g_{ce} \\ g_{de} & g_{ae} & g_{eb} & g_{ec} & \delta_{de} + 4\delta - i\Gamma_e \end{pmatrix} \begin{pmatrix} d(t) \\ a(t) \\ b(t) \\ c(t) \\ e(t) \end{pmatrix}, \tag{48}$$

where Γ_m is the decay rate of phonon mode m.

B. Fitting routine

For each value of the modulation depth shown in Fig. 3**e** of the main text, we measured the phonon populations of the three phonon modes a, b, and c for 71 equidistant values of δ between $\pm 2\pi \cdot 140\,\mathrm{kHz}$ and 100 values of τ_{BS} up to $50\,\mu$s. We numerically solve Eq. (48) and then fit the result to the measured data. Fig. 6 shows an example of a dataset and the corresponding fit. We take into account the infidelity of our SWAP and readout operations by matching the initial phonon populations to the measured populations at $\tau_{\mathrm{BS}} = 0$ and subtracting the readout infidelity of the qubit g-state of 6%. We fit all four relative phonon shifts δ_{md} and the couplings between modes a, b, and c presented in Fig. 3 of the main text, namely g_{ab}, g_{bc}, and g_{ac}. To both reduce the fit time and avoid overfitting the data, all other couplings are set equal to the value expected from Eq. (4) of the main text. We repeat this for 23 different values of the modulation depth, yielding the data presented in Fig. 3**e** of the main text.

To estimate how resilient our fit is against variation of the resulting parameters, we compute the residuals (sum of squared differences between the solution of the EOMs and the data points) when varying each parameter individually. We then find the value that increases those residuals by 5% in each direction and use that value as error bar in Fig. 3**e** of the main text.

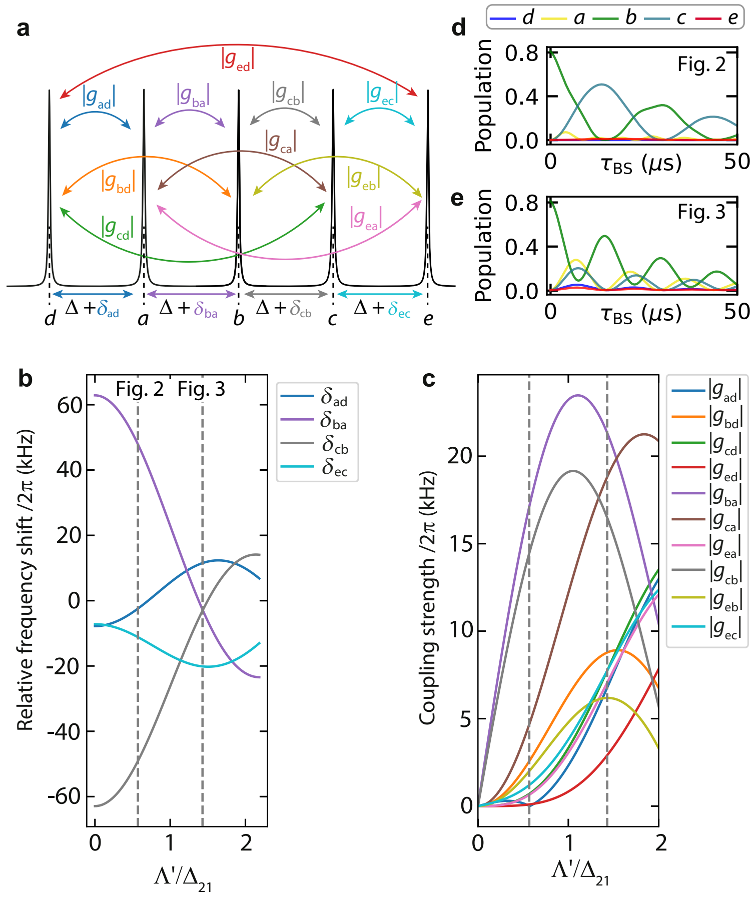

FIG. 5. **Characterization of the beam-splitter interaction between different acoustic modes.** **a** Schematic representation of the beam-splitter coupling between the five modes taken into account in our theoretical model. **b** Relative frequency shift between adjacent phonon modes for different values of Λ'/Δ_{21} according to Eq. (34). Vertical gray dashed lines indicate the operating points of the experiment in Fig. 2 and 3 of the main text. **c** Beam-splitter coupling strength between phonon modes as labeled in panel **a** according to according to Eq. (37). The couplings $|g_{ba}|$, $|g_{cb}|$ and $|g_{ca}|$ are the main coupling strengths also shown in Fig. 3 of the main text alongside value extracted from experimental data, whereas all the other couplings are considered spurious couplings as they are much lower in magnitude. **(d)** Solutions of EOMs at the operating point corresponding to Fig. 2 of the main text, including the populations of modes d and e. **(e)** Like (d), but for the second operating point corresponding to Fig. 3.

C. Spurious coupling to additional phonon modes

Here, we show how much spurious coupling we expect from the modes explored in the experiments in the main text. We consider two aspects of those couplings, namely the expected coupling strengths and the detuning from the resonance conditions for those spurious couplings during the experiments shown in the main text. From our derivation in Section IV, we get expressions for both the beam-splitter detunings (Eq. (34)) and the coupling strengths (Eq. (37)). In Fig. 5, we plot the resulting values. Fig. 5a shows a diagram of the five modes we consider with all-to-all coupling strengths and the detunings between adjacent modes in the rotating frame of the EOMs in Eq. 48. For example, g_{ad} labels the coupling strength between modes a and d, and δ_{ad} labels the detuning from the resonance condition of this coupling due to normal mode shifts of the modes a and d. The detunings between adjacent modes are shown in Fig. 5b and the couplings strengths are shown in Fig. 5c.
We distinguish two operating points, the first one at $\Lambda'/\Delta_{21} = 0.61$ corresponds to Fig. 2 of the main text, the second one, $\Lambda'/\Delta_{21} = 1.43$ corresponds to Fig. 3. At each point, we now consider how strong spurious couplings are and how far they are detuned from their resonance condition. For the first operating point, we only want to couple modes b and c. The highest unwanted coupling is g_{ab}, similar in strength to $|g_{cb}| = 2\pi \cdot 15.6\,\text{kHz}$ (purple and gray lines in Fig. 5c). However, its resonance condition is detuned from that of the intended coupling by $\delta_{ba} - \delta_{cb} \approx 2\pi \cdot 100\,\text{kHz}$. For $g_{\text{BS}}/\delta \ll 1$ we can approximate the the population transferred to mode a through this off-resonant coupling as the steady-state population of an off-resonantly driven two-level system, $(g_{\text{BS}}/\delta)^2 = 2.4\%$. This is on the same

order of magnitude as the off-resonant JC-interaction with the qubit $(g_b/\tilde{\Delta}_b)^2 = 5.4\%$. The other spurious couplings involving mode b at this operating point, g_{bd} (orange) and g_{eb} (olive) are much weaker ($\sim 2\pi \cdot 2.5\,\mathrm{kHz}$) with their resonance condition mismatched by similar detunings of $\sim 2\pi \cdot 100\,\mathrm{kHz}$, resulting in negligible infidelities. Both effects are much smaller than the phonon decay. During a $16\,\mu$s SWAP between modes b and c (main text Fig. 2d) the phonon loss including Purcell decay through the qubit is approximately 30%.

Next, we look at the second operation point, where we couple the three modes a, b, and c. The intended couplings are $g_{ba} \approx g_{cb} \approx g_{ca} \approx 2 \cdot 19\,\mathrm{kHz}$ (purple, gray, and brown in Fig. 5). All other unintended couplings between those three modes and the adjacent modes d and e have coupling strengths $\sim 2 \cdot 7\,\mathrm{kHz}$. The detuning of the resonance conditions of those couplings from that of the intended coupling is approximately $|\delta_{ad,ec} - \delta_{ba,cb}| \approx 16\,\mathrm{kHz}$. As those unwanted coupling strengths are no longer small compared to the detunings from their resonance conditions, we solve the EOMs in Eq. (48) using the analytical parameters given by Eq. (34) and (37) for the phonon populations at various beam-splitter times. We plot the resulting populations in Fig. 5d and e for the two operations points corresponding to main text Figures 2 and 3, respectively. We find that for the case of two-phonon coupling (Fig. 5d) the populations in the adjacent phonon modes d and e are negligible and the population in mode a reaches 8% around $4\,\mu$s and quickly returns to the modes of interest. In the case of three-phonon coupling (Fig. 5e), modes d and e reach populations of $< 5\%$.

In conclusion, we investigated the contributions of spurious coupling to additional modes and, while they are much smaller than the effects we want to demonstrate, we find that they play a non-negligible role. With improving device parameters, especially with larger qubit-phonon coupling strengths g_m, we expect this to improve. For example, the ratio of the unintended couplings to their detunings can be held constant (both detuning and coupling scale with g_m^2) while the targeted coupling increases.

VIII. BRIGHT AND DARK STATE HYBRIDIZATION

In the main text we present an experiment where three phonon modes are resonantly coupled via effective beam-splitter interactions (main text Fig. 3). When measuring the populations in each mode for different interaction times with the three couplings approximately on resonance (i.e. $\Delta_{ab} = \Delta_{bc} = \Delta_{21}$ and $\Delta_{ac} = 2\Delta_{21}$), we observe a typical multi-mode phenomenon, namely the coupling of one mode (in this case mode b) to a hybridized state of the two other modes. However, if we introduce a small detuning δ_{BS} to this resonance condition by slightly changing Δ_{21}, we observe an asymmetry between the resonance conditions of the coupling between mode a and b and that of b and c. Note that this is a different effect from the phonon frequency shifts described by Eq. (38). In this section we treat the case where the phonon modes are detuned from each other by equal amounts, but where the finite coupling between modes a and c introduces a normal mode splitting between the phonon modes, modifying the resonance conditions.

We start from a system of three harmonic modes a, b, and c at different frequencies, pairwise coupled via a beam-splitter interaction of the type derived in section IV. Mode pairs (a, b) and (b, c) are coupled via neighboring sidebands of the modulated qubit and mode pair (a, c) is coupled via next-to-neighboring sidebands. The beam splitter couplings, in the rotating frame of the three phonon modes, are detuned from their resonance conditions by δ_{BS} for neighboring sidebands and $2\delta_{\mathrm{BS}}$ for next-to-neighboring sidebands, described by the Hamiltonian

$$H_{\mathrm{rotating}} = g_{ab} b^\dagger a e^{i\delta_{\mathrm{BS}} t} + g_{bc} b^\dagger c e^{-i\delta_{\mathrm{BS}} t} + g_{ac} a^\dagger c e^{-2i\delta_{\mathrm{BS}} t} + \mathrm{h.c.} \tag{49}$$

The energy level diagram of this three-level system is depicted in the lab frame in Fig. 7**a** and in the rotating frame of the three phonon modes in Fig. 7**b** and **c**. The close to resonant coupling g_{ac} leads to a hybridization of modes a and c, with the hybridized modes $B = (ce^{-i\delta_{\mathrm{BS}} t} + ae^{i\delta_{\mathrm{BS}} t})/\sqrt{2}$ and $D = (ce^{-i\delta_{\mathrm{BS}} t} - ae^{i\delta_{\mathrm{BS}}} t)/\sqrt{2}$. Here, B is the hybridized mode arising from the coupling between c and a, which we refer to as the bright mode since it couples to mode b. On the other hand, D is referred to as dark mode as b does not directly couple to it. Furthermore, in our experiment, $g_{ac} \approx g_{bc} \equiv g_{\mathrm{BS}}$. Thus, we can rewrite the Hamiltonian using the hybridized modes, leading to

$$H_{\mathrm{rotating}} = \sqrt{2} g_{\mathrm{BS}} (b^\dagger B + b B^\dagger) + g_{ac} (B^\dagger B - D^\dagger D) \; . \tag{50}$$

Here we can see that if modes c and a were not coupled, mode b resonantly couples to a superposition of the two. In our case, however, $g_{ac} \neq 0$ and the second term in Eq. (50) does play a role. Noting that this second term is diagonal in the basis of B and D, we can unveil its effects by entering the rotating frame of the bright and dark modes via the transformation $U_{\mathrm{BD}} = \exp\left[-i g_{ac} (B^\dagger B - D^\dagger D) t\right]$. In this frame the Hamiltonian is approximately transformed to

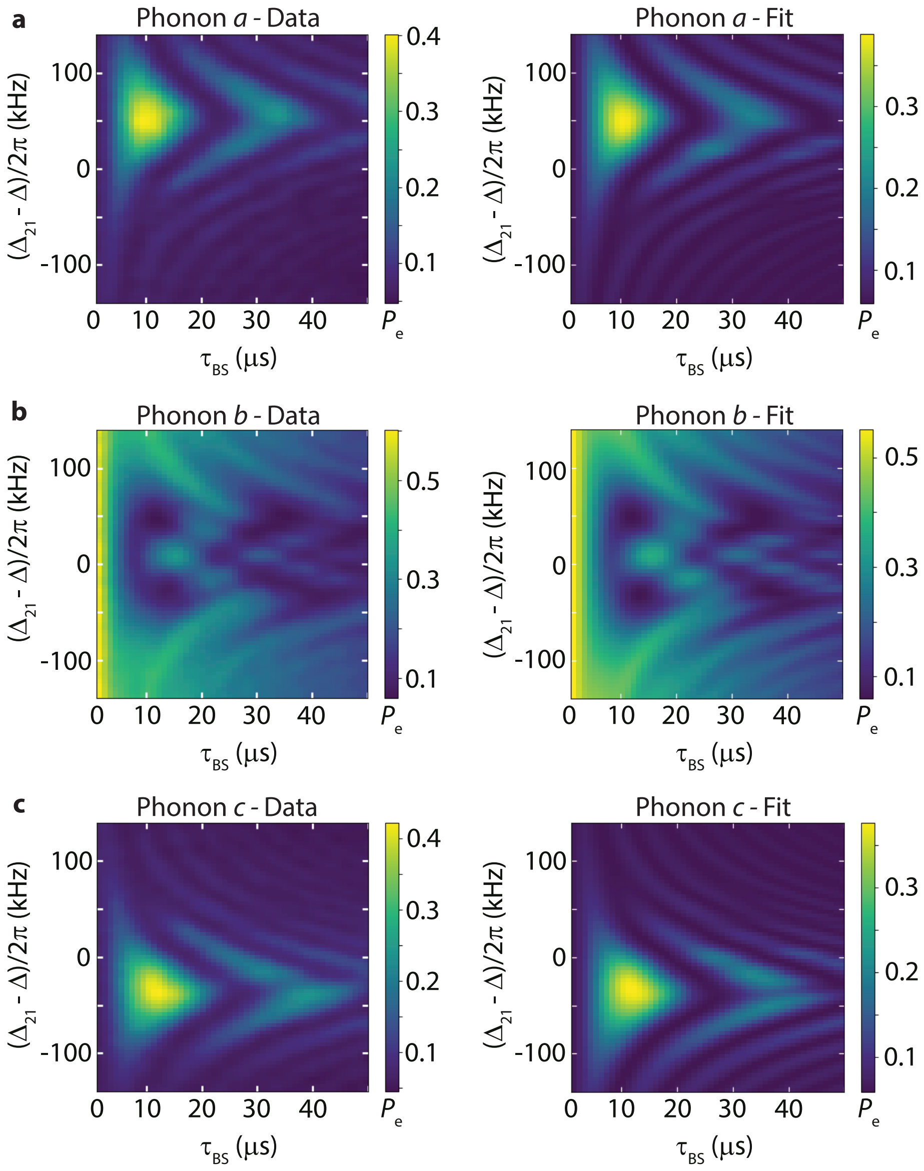

FIG. 6. **Example of three-mode coupling data and corresponding fit.** Phonon population measured and fit result for **a** Phonon mode a, **b** Phonon mode b and **c** Phonon mode c. The fit results for this particular dataset are $\delta_{ba} = 2\pi \cdot 48$ kHz, $\delta_{cb} = -2\pi \cdot 31.7$ kHz, $g_{bc} = 2\pi \cdot 17.2$ kHz, $g_{ab} = 2\pi \cdot 20.5$ kHz, and $g_{ac} = -2\pi \cdot 9.0$ kHz.

$$H_{\rm BD} = \sqrt{2} g_{\rm BS}(b^\dagger B e^{-ig_{ac}t} + bB^\dagger e^{ig_{ac}t}) = g_{\rm BS} b^\dagger \left(c e^{-i(\delta_{\rm BS}+g_{ac})t} + a e^{i(\delta_{\rm BS}-g_{ba})t} \right) + \text{h.c.} \tag{51}$$

This second rotating frame transformation is exact for $\delta_{\rm BS} = 0$. However, when $\delta_{\rm BS} \neq 0$, B and D include a time dependence leading to higher order terms in $H_{\rm BD}$, which we neglect here. From the first part of Eq. (51) we can see the behavior for $\delta_{\rm BS} = 0$, namely a coupling between b and B, which is detuned by g_{ac}, as drawn in Fig. 7**b**. In our data, this effect is visible through the reduced contrast of the Rabi oscillations of phonon mode b compared to modes a and c, (cf. main text Fig. 3**d**). More specifically, this shows how an excitation flows from mode b to both a and c and then back to b. From the second part of Eq. (51) it becomes clear that the coupling between modes b and c has a different resonance condition than that between modes b and a, which explains the asymmetry with respect to $\delta_{\rm BS}$ (or equivalently $\Delta_{21} - \Delta$ as labelled in Fig. 3**b** and **c** of the main text). We emphasize that this effect arises purely

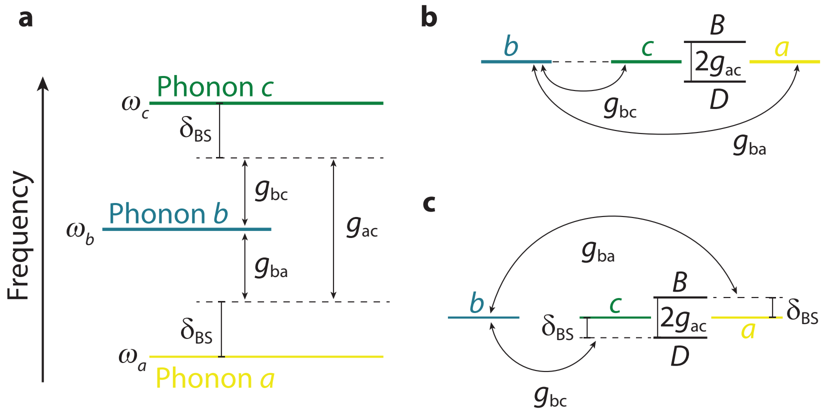

FIG. 7. **Energy level diagrams of of three coupled modes. a** Energy diagram in the lab frame with beam-splitter coupling detuned from the resonance conditions by $\delta_{\rm BS}$. **b** The system in the rotating frame of modes a, b, and c, as given by $H_{\rm rotating}$, Eq. (49) with $\delta_{\rm BS} = 0$. and **c** Same as **b** with $\delta_{\rm BS} = g_{ac}$.

from the non-negligible coupling g_{ac}, and is illustrated in Fig. 7**c** for the case of $\delta_{\rm BS} = g_{\rm ac}$.

[1] von Lüpke, U. *et al.* Parity measurement in the strong dispersive regime of circuit quantum acoustodynamics. *Nature Physics* **18**, 794–799 (2022).
[2] Bild, M. *et al.* Schrödinger cat states of a 16-microgram mechanical oscillator. *arXiv:2211.00449* (2022).
[3] Frattini, N. *et al.* 3-wave mixing josephson dipole element. *Applied Physics Letters* **110**, 222603 (2017).
[4] Zhang, Y. *et al.* Engineering bilinear mode coupling in circuit QED: Theory and experiment. *Physical Review A* **99**, 012314 (2019).
[5] del Pino, J., Slim, J. J. & Verhagen, E. Non-hermitian chiral phononics through optomechanically induced squeezing. *Nature* **606**, 82–87 (2022).
[6] Schuster, D. *et al.* AC Stark shift and dephasing of a superconducting qubit strongly coupled to a cavity field. *Physical Review Letters* **94**, 123602 (2005).
[7] Strand, J. D. *et al.* First-order sideband transitions with flux-driven asymmetric transmon qubits. *Physical Review B* **87**, 220505(R) (2013).
[8] Naik, R. K. *et al.* Random access quantum information processors using multimode circuit quantum electrodynamics. *Nature Communications* **8** (2017).
[9] Koch, J. *et al.* Charge-insensitive qubit design derived from the Cooper pair box. *Physical Review A* **76**, 1–19 (2007).
[10] Gao, Y. Y. *et al.* Entanglement of bosonic modes through an engineered exchange interaction. *Nature* **566**, 509–512 (2019).
[11] Xiao, Z. *et al.* Perturbative diagonalization for time-dependent strong interactions. *Physical Review Applied* **18**, 024009 (2022).
[12] Gely, M. F. & Steele, G. A. Superconducting electro-mechanics to test Diósi–Penrose effects of general relativity in massive superpositions. *AVS Quantum Science* **3**, 035601 (2021).
[13] Kjaergaard, M. *et al.* Programming a quantum computer with quantum instructions. *arXiv:2001.08838* (2020).
